# DNA metabarcoding data unveils invisible pollination networks

**DOI:** 10.1038/s41598-017-16785-5

**Published:** 2017-12-04

**Authors:** André Pornon, Christophe Andalo, Monique Burrus, Nathalie Escaravage

**Affiliations:** 1 Laboratoire Evolution and Diversité Biologique EDB, Université Toulouse III Paul Sabatier, F-31062 Toulouse, France; 2 CNRS, EDB, UMR 5174, F-31062 Toulouse, France

## Abstract

Animal pollination, essential for both ecological services and ecosystem functioning, is threatened by ongoing global changes. New methodologies to decipher their effects on pollinator composition to ecosystem health are urgently required. We compare the main structural parameters of pollination networks based on DNA metabarcoding data with networks based on direct observations of insect visits to plants at three resolution levels. By detecting numerous additional hidden interactions, metabarcoding data largely alters the properties of the pollination networks compared to visit surveys. Molecular data shows that pollinators are much more generalist than expected from visit surveys. However, pollinator species were composed of relatively specialized individuals and formed functional groups highly specialized upon floral morphs. We discuss pros and cons of metabarcoding data relative to data obtained from traditional methods and their potential contribution to both current and future research. This molecular method seems a very promising avenue to address many outstanding scientific issues at a resolution level which remains unattained to date; especially for those studies requiring pollinator and plant community investigations over macro-ecological scales.

## Introduction

As a consequence of the ongoing global changes, a dramatic and parallel worldwide decline in pollinators and animal-pollinated plant species has been observed^1^. Understanding the responses of pollination networks to these declines is urgently required to diagnose the risks the ecosystems may incur as well as to design and evaluate the effectiveness of conservation actions^2^. Early studies on animal pollination dealt with simplified systems, i.e. specific pairwise interactions or involved small subsets of plant-animal communities. However, the impacts of disturbances occur through highly complex interaction networks^3^ and, nowadays, these complex systems are currently a major research focus. Assessing the true networks (determined by ecological process) from field surveys that are subject to sampling effects still provides challenges^4^.

Recent research studies have clearly benefited from network concepts and tools to study the interaction patterns in large species assemblages^5^. They showed that plant-pollinator networks were highly structured, deviating significantly from random associations^6^. Commonly, networks have (1) a low connectance (the realized fraction of all potential links in the community) suggesting a low degree of generalization; (2) a high nestedness (the more-specialist organisms are more likely to interact with subsets of the species that more-generalist organisms interact with) the more specialist species interact only with proper subsets of those species interacting with the more generalist ones^7^; (3) a cumulative distribution of connectivity (number of links per species, *s*) that follows a power or a truncated power law function^8^ characterized by few supergeneralists with more links than expected by chance and many specialists; (4) a modular organization. A module is a group of plant and pollinator species that exhibits high levels of within-module connectivity, and that is poorly connected to species of other groups^9^.

The low level of connectivity and the high proportion of specialists in pollination networks contrast with the view that generalization rather than specialization is the norm in networks^10,11^. Indeed, most plants species are visited by a diverse array of pollinators which exploit floral resources from a wide range of plant species^12,13^. A main cause evoked to explain this apparent contradiction is the incomplete sampling of interactions^14^. Indeed, most network properties are highly sensitive to sampling intensity and network size^6^. Network studies are basically phytocentric i.e. based on the observations of pollinator visits to flowers. This plant-centered approach suffers nevertheless from inherent limitations which may hamper the comprehension of mechanisms contributing to community assembly and biodiversity patterns. First, direct observations of pollinator visits to certain taxa such as orchids are often scarce^15^ and rare interactions are very difficult to detect in field in general^2–5^. Pollinator and plant communities usually are composed of few abundant species and many rare species that are poorly recorded in visit surveys^16,17^. These rare species appear as specialists, whereas in fact they could be typical generalists. Because of the positive relationship between interaction frequency (*f*) and connectivity (*s*), undersampled interactions may lead to overestimating the degree of specialization in networks^18^. Second, network analyses have mostly operated at species levels. Networks have very rarely been up scaled to the functional groups or down scaled to the individual-based networks^19^, and most of them have been focused on one or two species only (but see ref.^20^ for an exception). The behavior of either individuals or colonies is commonly ignored, although it may influence the structure of the species networks^19^. Species accounted as generalists in species networks could, therefore, entail cryptic specialized individuals or colonies. Third, flower visitors are by no means always effective pollinators as they may deposit no conspecific pollen and/or a lot of heterospecific pollen^21,22^. Animal-centered approaches based on the investigation of pollen loads on visitors and plant stigmas may be more efficient at revealing plant-pollinator interactions^21,22^. However, the pollen identification requires considerable time and skills and is often limited to the genus or the family levels^23^. Altogether, these limits preclude investigating diverse communities in a wide range of habitats or throughout large temporal scale.

In this study, we test the potential of metabarcoding data to build plant-pollinator networks. Metabarcoding uses high-throughput DNA sequencing to identify taxa from mixed DNA samples^24^. It has been used to characterize the composition and/or the relative abundance of pollen in honey^25^ and on pollinator’s bodies^23,26–30^. However, to the best of our knowledge, no study has yet compared field surveys and metabarcoding results nor investigated the influence of molecular data on structural parameters of pollination networks^31^. From this perspective, we specifically ask two main questions: (1) how does metabarcoding compare to visit surveys to detect links in pollination networks; (2) what is the influence of metabarcoding data compared to data obtained from visit surveys on pollination network structure? Metabarcoding could actually provide a different picture of pollination networks if (i) it exhibits greater taxonomic sensitivity relative to traditional methods as previously shown in other systems^29^; (ii) it reveals hidden links^31^; (iii) these unveiled links change the connectivity pattern in the networks.

During a previous study^30^, we recorded the visits of 402 insects to plant species in subalpine *Rhododendron ferrugineum* heathlands (Central Pyrenees, southern France). The insects were then captured and the pollen they transported was identified, using metabarcoding. Metabarcoding results have been amply discussed in ref.^30^. Here, we use the formerly obtained visit and metabarcoding data to build and compare bipartite plant-pollinator networks (hereafter named N_obs_ and N_seq_ respectively) at three scales of resolution: (1) group-group networks (*gp-gp* N_obs_ and *gp-gp* N_seq_) accounting for interaction between groups of pollinators and groups of plants based on the flower morphology and reward accessibility; (2) pollinator species-plant species networks (*sp-sp* N_obs_; *sp-sp* N_seq_); (3) individual insects-plant species networks (*i-sp* N_seq;_ *i-sp* N_obs_).

## Results

### Plant-insect group networks

Diptera, mainly Empididae (24% of the 402 insects captured) and Syrphidae (20%), were the most abundant pollinators (47% of all visits). Bees (40% of all species), especially Bumblebees (26%), were also important components of the pollinator assemblages.

**Figure 1:**
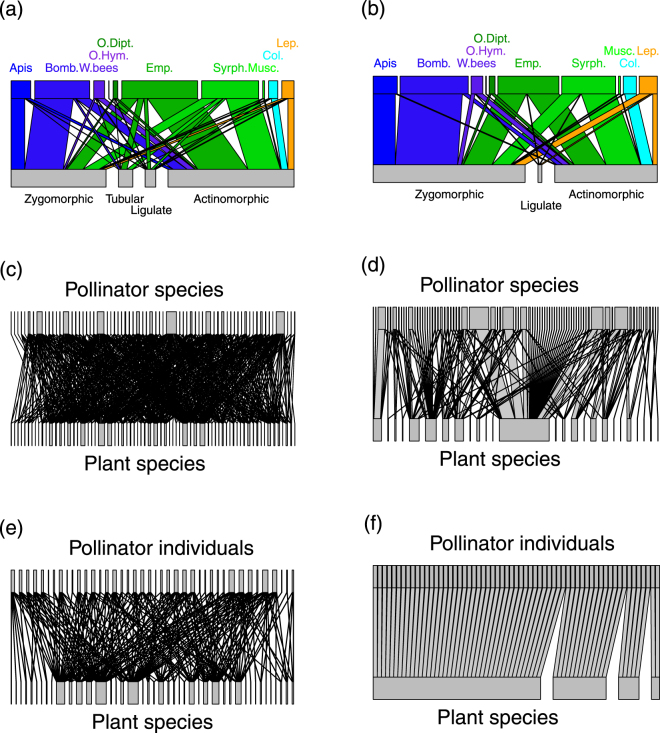
Bipartite pollination networks built from visit surveys (N_obs_, right panels) and metabarcoding (N_seq_, left panels) data. (**a**,**b**) Plant-pollinator groups; (**c**,**d**) plant-pollinator species; (**e**,**f**) individual pollinator-plant species (*Empis leptempis pandellei* as an example of pollinator species). Line thickness highlights the proportion of interactions. Apis: *Apis mellifera*; Bomb.: *Bombus sp*.; W.bee: wild bees; O.Hym.: other Hymenoptera; O.Dipt.: Other Diptera; Emp.: Empididae; Syrph.: Syrphidae; Col.: Coleoptera; Lep.: Lepidoptera; Musc.: Muscidae.

Overall, metabarcoding provided insight on interactions between plant and insect groups (Fig. 1a) fully consistent with the visit survey (Fig. 1b): Diptera interacted with open flowers (actinomorphic), often yellow for Syrphidae, whereas bees interacted with zygomorphic-shaped flowers. However, bees appeared less specialized upon zygomorphic flowers in the *gp-gp* N_seq_ as they visited more frequently actinomorphic flowers (32% of bee visits against 14% in *gp-gp* N_obs_). In contrast, Empididae interacted less frequently with zygomorphic flowers in *gp-gp* N_seq_ (21% of Empididae visits against 47% in *gp-gp* N_obs_).

From the plant point of view, in both *gp-gp* N_obs_ and *gp-gp* N_seq_, zygomorphic flowers received principally visits from bees (≈57%) and Diptera (≈35%). About two thirds of actinomorphic flowers were visited by Diptera, while these flowers were less visited by bees (≈21% of actinomorphic visits). Finally, there is a clear asymmetry in some plant-pollinator interactions: bees depend more strongly on zygomorphic flowers than the latter depend on bees, although this trend was attenuated in *gp-gp* N_seq_. No asymmetry was observed for the main Diptera groups (Empididae and Syphidae).

### Pollinator species-plant species networks

Null models analyzes showed that, for most parameters (except for nestedness, module amount, extreme plant and insect specialization in *sp-sp* N_obs_ and *sp-sp* N_seq_ and mean insect specialization in *sp-sp* N_seq_) both *sp-sp* N_obs_ and *sp-sp* N_seq_ differed from their respective null networks (Table 1). Thus, both network types appeared to be principally structured by ecological rather than by random processes. On the one hand, both *sp-sp* N_obs_ and *sp-sp* N_seq_ shared several characteristics (Table 1): low connectance and high nestedness, the same set of outlier species which concentrate the bulk of interactions, among the pollinators (*Sphaerophoria batava*, *S*. *infuscata*, *S*. *scripta*, *Volucella bombylans*, *Empis leptempis pandellei*, *E*. *euempis tessellata*, *Apis mellifera*, *Bombus lucorum*, *B*. *wurflenii*, *B*. *pascuorum*, *Lasioglossum albipes*, *Oedemera virescens*) and the plants (*R*. *ferrugineum*).

**Table 1:** Characteristics of species networks built from visit survey (*sp-sp* N_obs_) and metabarcoding data (*sp-sp* N_seq_).

	*sp-sp* N_obs_	95% CI	*sp-sp* N_seq_	95% CI
No. insect species (A)	76		66	
No. plant species (P)	26		68	
Network size (A × P)	1976		4488	
No. links (I)	153* a	197–214	612* b	652–689
Connectance (C = I/A×P)	0.077* a	0.099–0.108	0.136* b	0.145–0.153
Nestedness (100−T)/100	0.96^ns^ a	0.95–0.97	0.92^ns^ b	0.91–0.94
Modularity (M)	0.44* a	0.17–0.28	0.29* b	0.12–0.15
Number of modules	7^ns^ a	5–9	5^ns^ a	4–6
Interaction density I/(A + P)	1.5* a	1.93–2.1	4.57* b	4.87–5.14
Mean plant linkage level (I/P)	5.88* a	7.58–8.23	9* b	9.59–10.13
Mean insect linkage level (I/A)	2.01* a	2.59–2.82	9.27* b	9.88–10.44
Interaction diversity (*H*_2_’)	0.47* a	0.12–0.17	0.18* b	0.085–0.10
Interaction evenness (*E*_2_ = *H*_2_”/*H*_*max*_)	0.56* a	0.63–0.65	0.69* b	0.718–0.723
Mean plant specialization index *d’*	0.51* a	0.20–0.36	0.22* b	0.13–0.18
Mean insect specialization index *d’*	0.26* a	0.17–0.24	0.17^ns^ a	0.145–0.2
Extreme plant specialization (%)	34.6^ns^ *a*	30.8–34.6	11.8^ns^ *b*	8.8–11.8
Extreme insect specialization (%)	60.53^ns^ *a*	53.9–61.8	10.6^ns^ *b*	10.6–12.1

CI: confidence interval calculated from null models. *Indicates that either the *sp-sp* N_seq_ or the *sp-sp* N_obs_ differed significantly from their corresponding null models. Indices that do not share the same letter differ significantly (P < 0.001) independently on the network size and sampling effort (P < 0.05 for italic letters). T: temperature.

**Figure 2:**
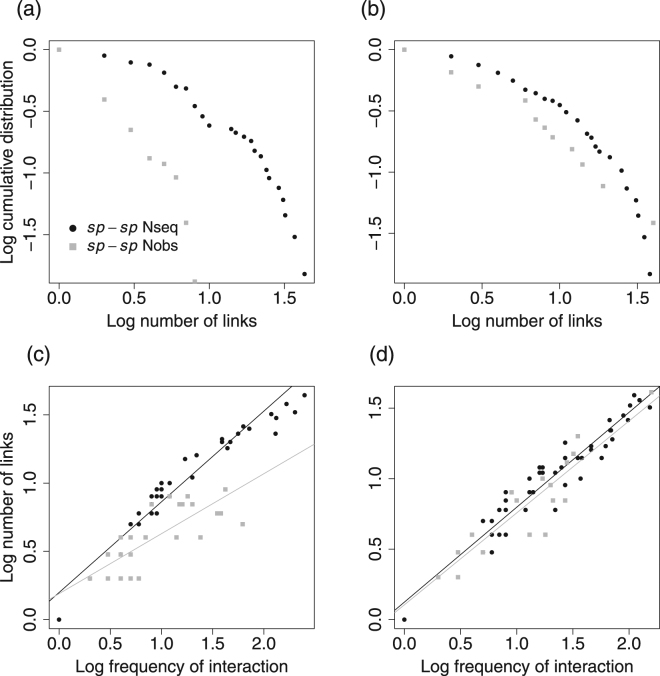
Connectivity pattern in species pollination networks obtained from visit surveys (*sp-sp* N_obs_) or metabarcoding (*sp-sp* N_seq_) data. (**a**,**b**) The cumulative distribution of mean number of links per pollinator and plant species respectively. (**c**,**d**) The relationships between interaction frequency and mean number of links per pollinator and plant species respectively.

On the other hand, when comparing *sp-sp* N_obs_ and *sp-sp* N_seq_, all structural parameters changed significantly (Table 1). First, metabarcoding data unveiled many hidden interactions (x 4) and provided a much denser *sp-sp* network (Fig. 1c,d): in the *sp-sp* N_obs_, 76 insect species interacted with 26 plant species whereas, in the *sp-sp* N_seq_, 66 insect species interacted with 68 plant taxa (57 species and 11 genera), one third of them growing outside the prospected areas and many of them being rare. Beside the aforementioned pollinator and plant species, *Empis euempis ciliate*, *Hippocrepis comosa*, *Lotus corniculatus*, *Conopodium majus*, *Potentilla erecta*, *Geranium sylvaticum*, *Genista pilosa*, *Ranunculus polyanthemoides*, *Cytisus scorparius*, *Thalictrum aquilegiifolium* were other species largely involved in *sp-sp* N_seq_ interactions. There were ten missing insect species in the *sp-sp* N_seq_ that yielded no or few sequences. In *sp-sp* N_seq_, most insect specimens had visited several plant species before being captured and consequently, extreme insect specialization (insect visiting only one plant species) was 6 times lower in *sp-sp* N_seq_ than in *sp-sp* N_obs_. Extreme plant specialization was also 3 times lower in *sp-sp* N_seq_. Second, compared to *sp-sp* N_obs_, *sp-sp* Nseq had higher connectance (x 1.77), interaction evenness (x 1.23) interaction density (x 3) and mean linkage level of plants (x 1.5) and pollinators (x 4.6) (Table 1). In contrast, modularity was lower in *sp-sp* N_seq_ but was consistent with the results of *gp-gp* N_seq_: Bees and zygomorphic flowers were in the same module, whereas Diptera and actinomorphic flowers were in other modules (see Supplementary Fig. S1). Also, some abundant Diptera were in separate modules (*S*. *scripta*, *V*. *bombylans*, *E*. *e*. *tessellata* against *E*. *l*. *pandellei*, *S*. *infuscata*, *S*. *batava*). Both plant and insects were more generalized in *sp-sp* N_seq,_ both at the network level (lower *H*_2_’ value) and at the species level (lower plant d’ and extreme specialization values) (Table 1). Third, the numerous interactions revealed by metabarcoding data were shared by a lower number of insect species in *sp-sp* N_seq_ compared to *sp-sp* N_obs_ (Table 1) and the connectivity pattern changed between networks. Indeed, although for both *sp-sp* N_seq_ and *sp-sp* N_obs_ the cumulative distribution shaped a truncated power law, the curve had a longer tail (a smaller proportion species with few links) and higher values (generalized species with more links; Fig. 2a) in *sp-sp* N_seq_. In contrast, the increased number of interactions in *sp-sp* N_seq_ spread out over a much larger number of plant species with a remarkably conserved pattern of plant connectivity between networks (Fig. 2b). In both *sp-sp* N_seq_ and *sp-sp* N_obs_ the mean number of links per pollinator (*sp-sp* N_seq_: slope = 0.664, P < 0.001; *sp-sp* N_obs_: slope = 0.439, P < 0.001; Fig. 2c) or plant species (*sp-sp* N_seq_: slope = 0.673, P < 0.001; *sp-sp* N_obs_: slope = 0.652, P < 0.001; Fig. 2d) increased with the interaction frequency. However, the slope of the pollinator regression lines was steeper (P < 0.001) in the *sp-sp* N_seq_ compared to *sp-sp* N_obs_ principally because of the presence of supergeneralized pollinators in *sp-sp* N_seq_. In contrast, both *sp-sp* N_seq_ and *sp-sp* N_obs_ had quite equal slopes of plant regression lines (P = 0.090).

### Insect individuals-plant species networks

Null models showed that, for most structural parameters (except for the module amount, and the extreme plant and insect specialization) the *i-sp* N_seq_ of all insect species were mainly determined by ecological rather than by random processes. The *i-sp* N_seq_ of the 11 most abundant pollinator species (Fig. 1e,f; see Supplementary Fig. S2 and Table S1) contained more plant species than the *i-sp* N_obs_ (17–43 *vs* 3–8 species respectively). The difference in the amount of plant species between *i-sp* N_seq_ and *i-sp* N_obs_ was particularly marked in supergeneralized insect species (*A*.*mellifera*, *B*. *lucorum*, *E*. *e*. *tessellata*, *E*. *e*. *pandellei*, *V*. *bombylans*) and weaker in more specialized species (*B*. *pascuorum*, *B*. *wurflenii*, *S*. *batava*, *S*. *infuscate*, *S*. *scripta*). Overall, *i-sp* N_seq_ had a larger size (133–1763 *vs* 39–328), more interactions (38–248 *vs* 7–61), higher density (1.46–3.57 *vs* 0.54–0.94) and evenness of interactions (0.70–0.80 *vs* 0.52–0.75) and higher nestedness (0.71–0.93 *vs* 0.5–0.79). Metabarcoding data tended to provide lower connectance and lower mean plant linkage levels than visual records for the supergeneralized insect species (see supplementary Table S1). In contrast, these parameters tended to be higher in *i-sp* N_seq_ for specialized species. Moreover, specialized species’ individuals seemed less specialized in *i-sp* N_seq_ (lower index *d’*) than in *i-sp* N_obs_, while changes were hardly perceptible for supergeneralized species’ individuals.

**Figure 3:**
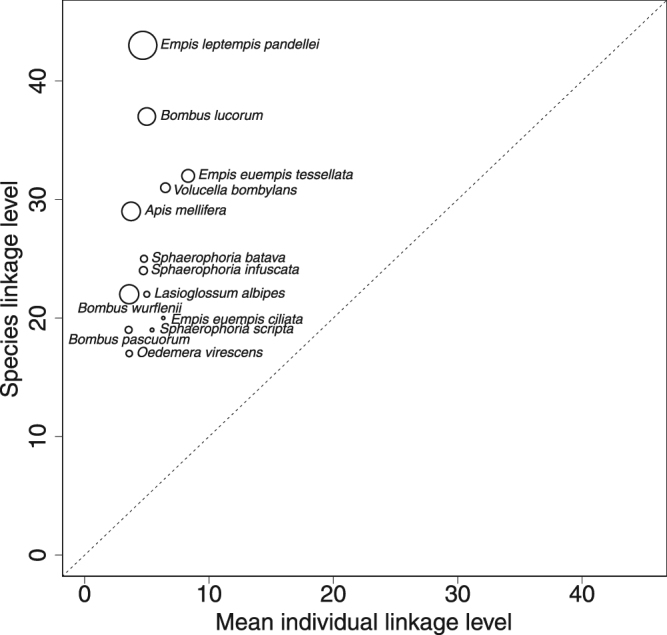
Asymmetry in mean individual and species linkage levels in networks obtained from metabarcoding data.

*I-sp* networks had a higher connectance than the *sp-sp* networks. This resulted principally from the smaller size of *i-sp* networks. Indeed, a higher specialization of individuals relative to the population they belonged to would have induced a lower conductance in *i-sp* networks. Actually, the *i-sp* N_seq_ harbored a lower interaction density (2.14 ± 0.64 SD) than the *sp-sp* N_seq_ (4.57) and a 4 to 6 times lower mean pollinator linkage level (Fig. 3). Even the specimens of supergeneralized species (*B*. *lucorum*, *E*.*l*. *pandellei*) appeared not to be more generalized than those of other species (Fig. 3).

## Discussion

### Pros and cons of metabarcoding relative to visit surveys

Given that most network metrics are influenced by network size and as metabarcoding has high sensitivity to small DNA amounts, contaminations could have inflated the number of interactions and species and given unrealistic networks accordingly^32^. Indeed, small contaminant DNA may generate false positives since it has the same, if not, higher probability of being amplified as the targeted DNA. In contrast, in our study, DNA extraction from pollen most likely yielded quantities of DNA far in excess of any potential laboratory contaminants with higher probability to be amplified. Moreover, DNA into pollen grain could have been well conserved. Still, we applied laboratory practices adapted to prevent sample contaminations when working with very small quantities of degraded DNA^30^. As a consequence, the risk of contamination was likely reduced in our study. However, contaminations could still have occurred in field from airborne pollen, pollen contamination among pollinators and non-pollen-plant materials. The most abundant plants in airborne pollen, *i*.*e*. wind-pollinated species, were not considered in the network analyses. Since the insect-pollinated plants are generally rare in airborne pollen, we consider this source of contamination to be likely negligible. Pollen cross contamination among pollinators was prevented by using new clean nets for each capture and placing each individual in separate clean scintillation tubes. Yet, an individual pollinator visiting a flower could have picked up heterospecific pollen deposited by other pollinators. However, until we know, this contamination source is limited although probably insufficiently investigated. For instance, only 0 to 5 heterospecific pollen grains (from different species) were found on the monolectic hairy bees *Hoplitis adunca* (*Panzer*) specialized on the highly generalist plant species *Echium vulgare* L.^14^. Eventually, we firmly believe that the application of the 1,000 sequence threshold has removed most of the potential above-described contaminations (including no-pollen plant particles). Indeed, this threshold was able to remove most of the potential background contamination generated by wind-pollinated grasses and animal-pollinated species as well. Indeed, grasses dominating the grasslands of the site have much higher contaminating potential than insect-pollinated species (see Methods). Nonetheless, we are aware that despite all precautions we took to prevent potential errors, metabarcoding could have inflated the size of N_seq_ and the interaction amounts in N_seq_ which are known to both affect most network metrics^6^. Then, to compare visit survey and metabarcoding structure we used a random sampling method consisting to generate rarefied N_seq_ matrices of the same size and sampling intensity as N_obs_ matrices. Moreover, we calculated that dividing or multiplying twice the 1,000 sequences threshold, although altering the number of species and interactions (for example, applying a 2,000 seq threshold led to a 20% reduction of the interaction amounts in *sp-sp* N_seq_; see Supplementary Table S2) did not fundamentally change the N_seq_ structure and not at all the general conclusions about the large difference between molecular-based *versus* visit-based networks, thus highlighting the robustness of our results.

Methodologies used to deal with PCR and sequencing errors and potential misidentifications are also detailed in ref.^30^. The most common explanations for misidentifications are the absence of the species in the reference library or the lack of species-level variation in sequences^33^. In contrast, the risk of misidentification is minimized when reference databases are established specifically for local assemblages, as in our study. The lack of species-level variation may lead to sequences being indistinctly assigned to two or more species. When this occurred in our study, the taxonomic identification was resolved at higher taxonomic level. Then, taxa assigned to a higher level than genus were discarded from the network analyses. Finally, misidentifications commonly occur among closely related species, typically belonging to the same genera. Actually, DNA barcoding can achieve a high rate of species discrimination for distantly related taxa^33^. As only three genera had more than one species (*Hieracium sp*. *Lathyrus sp*. *Ranunculus sp*.)^30^, we believe that misidentification was low in our study.

On the other hand, metabarcoding has numerous potential advantages relative to traditional methods: (1) it is becoming increasingly established that metabaroding improves palynology diversity assessments in comparison with traditional optical microscopy especially in large and diverse pollen samples^29,31^. This occurs through identification of novel taxa of and better possibilities to distinguish species within a genus^34,35^; (2) it allows investigating the entire insect pollen load which is hardly possible through pollen identification^14^; (3) on contrary to visit observations, it provides information about what pollinator transports which pollen; (4) it provides record of foraging pattern across time as pollen accumulates on insect bodies^36^ especially in species with low grooming activity such as Syrphidea^37^; this multispecies pollen accumulation can moderate the impression of specialization arising from visitor constancy^37^ or the basking of flies on flowers^38^; (5) it depicts interactions occurring outside the investigated area. In consequence of (4) and (5), metabarcoding data considerably enlarges the spatiotemporal window observation of plant-pollinator interactions allowing, in our study, to depict much more plants species and interactions than the visit surveys; (6) it significantly reduces the proportion of rare interactions for both rare and supergeneralized species, with substantial consequences on specialization estimation and connectivity patterns; (7) finally, like the identification of pollen through microscopy but contrary to visit surveys, it allows downscaling networks from the species level to an individual one.

### The potential of metabarcoding data for investigating plant-pollinator networks

#### Plant-insect group networks

As has been observed in other cold habitats^38,39^, Diptera, mostly Empididae and Syrphidae are major pollinators in our study site. Bees and particularly bumblebees are known to be important pollinators of the studied communities, especially for the shrub *R*. *ferrugineum* ^40^. Both metabarcoding and visit surveys provided concurring insights on interactions between plant and insect groups (*gp-gp* N_seq_ and *gp-gp* N_obs_) fully consistent with the literature data: *Diptera* preferred simpler shaped actinomorphic flowers with exposed rewards, mainly yellow for Syrphidae^41,42^ whereas bees interacted preferentially with complex zygomorphic flowers^37^. Accordingly, in *sp-sp* N_seq_, bees and zygomorphic flowers were in the same module, whereas Diptera and actinomorphic flowers were in other modules. The lack of accurate data about plant and insect traits did not allow us to comprehend why closely related and visibly very similar Diptera species (*Empis sp*.*; Sphaerophoria sp*.) were found in different interaction modules. On the other hand, metabarcoding data suggested that bees were relatively less specialized upon zygomorphic flowers and the Diptera Empididae relatively more specialized upon actinomorphic flowers in metabarcoding-based network compared to visit survey network. In addition, many Empididae visits to zygomorphic flowers did not apparently translate in pollen transport in *gp-gp* N_seq_ possibly because anthers hidden inside the corolla were not accessible to these insects. Therefore the relative barrier of zygomorphic floral structure to interaction with Empididae seemed stronger in *gp-gp* N_obs_ compared to *gp-gp* N_seq_. Similarly, the ten missing insect species in the *sp-sp* N_seq_ that yielded no or few sequences possibly carried either no or few pollen grains, thus being ineffective pollinators^21,22^ despite visiting the flowers. For example, *Bistorta officinalis* received 92% of *E*. *e*. *tessellata’s* visits in *sp-sp* N_obs_ but only 11.5% in *sp-sp* N_seq_. This may account for the large differences between pollen transport network and visitation networks revealed in other studies^14,43,44^. Otherwise, *sp-sp* N_obs_ were found to have features similar to those of other cold environments^39,45^ and both *sp-sp* N_obs_ and *sp-sp* N_seq_ shared several characteristics common to most pollination networks^6,7,9^: low connectance and high nestedness, the same plant and insect species which concentrate the bulk of interactions.

#### Pollinator species-plant species networks

Contrary to previous studies that compare networks based on pollen analysis with visit survey networks^14,43^, we detected many more plant species and interactions with metabarcoding than with visit surveys. This resulted in very important changes in all network structural parameters. On the contrary, Bosch *et al*.^14^ found not significant changes in some essential parameters as the interaction amount, the connectance, the mean plant and animal connectivities, the plant and pollinator connectivity patterns. Moreover, they found higher nestedness and modularity in the pollen network than in the visit network while we found the opposite. Unlike us, Popic *et al*.^43^ found that pollen network had smaller size and interaction amount and a higher specialization compared to the visit network, principally because many insects which had visited plants did not carry pollen.

These different findings could have several causes: (1) in our study, all pollen grains were retrieved by washing insects which would have been difficult to achieve by dabbing bodies with cubes of fuchsin gel^14,43^; (2) the impossibility to investigate all pollen grains in densely populated slides^14^; (3) the higher taxonomic sensitivity of metabarcoding relative to traditional methods^29^; (4) the inflation of the interaction amounts by the metabarcoding.

As a consequence of the larger number of species and interactions, the connectance, the interaction evenness and the connectivity all increased greatly in the *sp-sp* N_seq_. Intrinsically, connectance exponentially increases with generalization and decreases with the number of species involved^32^. While the *sp-sp* N_seq_ was more diversified, the higher connectance in this network primarily resulted from a much higher plant and insect generalization visible both at the network level (lower *H*_2_’ value) and at the species level (lower d’ and extreme specialization values). This occurred because rare plant species (for instance *Thymus serpyllum*, *Trollius europaeus*, tubular species) undetected during visit surveys, were found in insect pollen loads and/or because rare insect species (for instance *Parasyrphus vittiger*, *Hylemya vagans*) observed to visit only one plant species carried the pollen of several other ones. This perfectly illustrates the tendency of visit surveys to dramatically confound specialization with rarity^44^ and also the fact that visual or genetic pollen analysis allows for the detection of interactions involving rare species^14^.The detection of rare interactions for rare but also for surpergeneralized species and of a higher number of plant species involved in the network significantly changed the connectivity pattern (i.e. the cumulative distribution of connectivity and relationship between the interaction frequency and the number of links per species). This was especially true for insects that interacted with many more plant species in *sp-sp* N_seq_ than in *sp-sp* N_obs_ more so than for plants for which no significant shift in connectivity pattern was observed. This suggests that metabarcoding data did not radically change the partitioning of interactions among plant species whereas new interactions in *sp-sp* N_seq_ concerned preferentially supergeneralized insect species. Therefore, these various network responses to molecular data highlighted that the shifts in network structure were not a mere straightforward consequence of new interactions detected by metabarcoding.

#### Insect individuals-plant species networks

Very few studies have investigated individual networks^19,20^. Our study differed from those because we investigated community-wide plant-pollinator networks for each single species. Congruent with the results obtained at species level, metabarcoding data changed dramatically the topology of *i-sp* network relative to visit survey data. Specifically, *i-sp* N_seq_ contained many more plant species and interactions, higher density and evenness of interactions and higher nestedness than the *i-sp* N_obs_. Interestingly, these changes did not affect individuals equally. Indeed, individuals of specialized species appeared to be more generalist (as shown by index *d’*) in *i-sp* N_seq_ than in *i-sp* N_obs_ whereas no important change was observed for supergeneralized species’ individuals. This finding suggests that field surveys struggle to detect interactions the specialized species’ individuals have outside their preferred plant species. The impact of rareness which tends to increase artificial specialization in networks^18,46^ cannot be evoked here, given that both *i-sp* N_obs_ and *i-sp* N_seq_ had almost equal individual number. Our results highlight the importance of building separate individual network for each pollinator species rather than building a global network including all individuals of all pollinator species^18^.

Congruent with other findings^18^, generalized pollinator species were composed of relatively more specialized individuals (*i-sp* N_seq_). This confirms that the behavioral plasticity of individuals in their food plant choice leads to a larger population niche breadth and shapes the structure of the *sp-sp* N_seq_ as a whole. In almost all pollination network studies published to date, nodes and links represent averages of characteristics of species and their interactions^47^. Since the level of organization at which interactions actually occur is individual, potentially important information may be lost by the process of averaging species data, thereby increasing the risk of misinterpreting the findings. For example in our study, the network constructed at the species level did not capture the specialization of individuals. Individual specialization may result from the intra-^19^ or inter-specific competition^48^ and/or may be viewed as a mechanism for the species to achieve a broader niche^20^. Since it may increase conspecific pollen deposition on the stigmas^49^ individual specialization may directly benefit plant reproduction^50^ and drive population dynamics and ecosystem functioning^51^. Downscaling networks from species to individuals and taking into account individual behaviors in pollination network is therefore essential to understand the mechanisms of community assembly and evolutionary processes^52^.

### Contribution of metabarcoding to current and future researches

By highlighting a higher level of connectivity, metabarcoding contributes to reducing the apparent paradox between the observed generalization considered as the norm in pollination systems^10,11^ and the higher level of specialization commonly estimated in networks. Visitation networks are commonly biased toward specialization of rare species because of the under-sampling of rare interactions^33^. Under-sampling species, rare interactions or examining interactions in too small an area can be a problem when attempting, for instance, to assess the beta-diversity of interactions or the phylogenetic structure of networks. Indeed, both are sensitive to sampling effort and between-site distance^53,54^. Actually, by detecting a larger number of species interactions over longer distances, metabarcoding may increase the area covered by the network potentially including less closely related plant species from various habitats. This may in turn decrease both interaction beta-diversity and the phylogenetic signal in networks. In a functional perspective, a higher generalization level can provide a higher potential for functional redundancy amongst species. Generalist and partially redundant networks may be more robust in the face of climate change^55^, species extinctions, invasive species^38^ or the impacts of habitat management^56^. Thus, the response of the pollination network to any kind of disturbances could be more accurately predicted from metabarcoding data than from visit surveys.

Unlike what was observed in the species-based network, a comparatively higher specialization occurred between plant and pollinator groups as well as between individual pollinators and plant species. The higher specialization in *gp-gp* networks concurred with the view that specialization can occur at a higher taxonomical level than the species level and seemed clearly related to the pollination syndrome^57^ and functional groups concepts^13^: the zygomorphic and actinomorphic flower groups have floral traits more adapted to bee and Diptera pollination respectively^36^. Thus, metabarcoding may be adapted in studying functional aspects of pollination networks especially in assessing the phenotypic matching between plant and pollinator species^53^.

Metabarcoding opens the path to perform parallel description of insect and stigmatic pollen loads, coupled with measures of pollinator effectiveness in depositing pollen^44^ onto stigma will give invaluable complementary data. Establishing the functional links between pollen-transport networks and pollen-deposition networks will nevertheless require more efforts and investigations. Whether in the near future this molecular method can provide quantitative information on plant-pollinator interaction (i.e. using the number of sequences as a direct measure of interactions or as a mean to categorize them as weak, medium and strong interactions) is still under consideration^30^. Such data would considerably improve our knowledge about the types and the magnitude of interactions between direct (plants-pollinators) or indirect (plants species through shared pollinators) partners.

Focusing efforts on exhaustive sampling of pollinators and using metabarcoding to identify pollen can be a very efficient mean to investigate plant-pollinator interactions and the structural topology of networks. Being faster and having higher taxonomic sensitivity than the traditional methods, metabarcoding^31,34^ should provide valuable opportunities to address many scientific outstanding issues at a resolution level never attained to date and requiring investigations of pollinator and plant communities over macro-ecological scales. Another limit to wide-range studies is the impossibility to identify many insect species in field. In this respect the accurate identification of pollinators with barcode CO1 fragments^31^ applied to solutions used to wash pollen from bodies (personal observations) should be very useful.

## Methods

Here we used data obtained from a previous study^30^. For pollen identification, short DNA markers (less than 250 bp) were used, namely the chloroplastic marker P6 loop of *trnL* (UAA) intron and the nuclear *ITS1* marker because the pollenkitt is composed of degraded, often multinucleate cells of the parent plant^58^, with possibly degraded DNA while the DNA into the pollen grain should be more preserved. The visitor sampling, the preparation of samples for the sequencing, the sequence analysis and plant taxon assignation as well as all precautions we took to prevent contaminations and species misclassification are detailed in ref.^30^.

Plant species-pollinator individuals matrices (M = [a_*ij*_] _*PxA*_; P: plants, A: pollinators) were completed with data either from field survey (M_obs_) or metabarcoding (M_seq_) and subsequent N_obs_ and N_seq_ networks were built. Only sequences belonging to insect-pollinated plant species growing either on the site or in the vicinity were kept in the matrices. The data obtained from the four communities were merged because they belonged to the same type of vegetation (*R*. *ferrugineum* heathlands). In matrices, a_*ij*_ was either a visit (1; 0 otherwise) of an individual insect *j* to the plant species *i* (N_obs_) or the presence of more than 1,000 *trnL* or ITS1 DNA sequences (1; 0 otherwise) of the plant species *i* in the pollen load of insect *j* (N_seq_). A 1,000 sequence threshold was applied to prevent, as far as possible, contaminations that could have risen for instance from airborne pollen or non-pollen plant tissues deposited on insect bodies. This threshold was based on previous findings^30^ and the analysis of traces (here sequences) left by grasses species on insect bodies in grasslands of the study site although this wind-pollinated plants were not considered in any network analyses. The rationale behind this approach was that, since insects move, rest and nest (e.g. *Bombus sp*.) in the grassland matrice and as the wind-pollinated plants are usually far more abundant in airborne pollen than insect-pollinated plants, grasses should represent a level of widespread background contamination that insect-pollinated plants would barely reach, if ever. In consequence, managing to remove grass contamination would, in all hypotheses, also remove potential contaminations from insect-pollinated plants. The observation that 35% of insects had no grass sequences and 60% less than 100 grass sequences suggested a relatively widespread although quite low (considering the number of PCR cycles applied) background contamination of insects by airborne pollen and/or non-pollen grass materials (see Supplementary Fig. 3). On the other hand, relatively few insects (11.5%) had more than 1,000 grass sequences (a maximum of 26,952 sequences) which may be attributed to occasional pollen foraging^59^ or the use of grass inflorescences by insects as landing areas. For insects with between 100 and 1,000 sequences we were not able to know if the sequences resulted from intentional visits *versus* contaminations by grasses. Consequently, as a precaution and in aim to prevent as far as possible potential contaminations, we took the count of more than 1,000 sequences from a given plant species as proof of link to that species. In doing so, we were able to remove 88.5% of grass sequences from the plant-pollinator matrices. We were confident that applying this 1,000 sequence threshold would thereby have removed virtually all background contamination from insect-pollinated plants. We are nevertheless aware that this step could have eliminated true interactions. For example, some insects (species or individuals) observed in N_obs_ but yielding less than 1000 sequences for any plant species were excluded from the N_seq_.

We built bipartite (1) group-group networks (*gp-gp* N_obs_ and *gp-gp* N_seq_) accounting for interaction between groups of pollinators (*Apis mellifera*, bumblebees, wild bees, other Hymenoptera, Syrphidae, Empididae, other Diptera, Coleoptera, Lepidoptera) and groups of plants based on the flower morphology and reward accessibility (zygomorphic < tubular actinomorphic (hereafter named tubular) < ligulate (Asteraceae) < actinomorphic); (2) pollinator species-plant species networks (*sp-sp* N_obs_; *sp-sp* N_seq_); (3) individual insects-plant species networks (*i-sp* N_seq;_ *i-sp* N_obs_) for the eleven most abundant insect species (see supplementary Fig S3 online for the species considered).

Both the visualization and the calculation of common parameters (Table 1) of N_seq_ and N_obs_ were performed using the R package ‘bipartite’ (R Development Core Team, 2013; version 1.17^60^. The connectance and the nestedness (100-T)/100 with T = temperature; ranging from 0 (chaos) to 1 (perfect nestedness) were calculated. *H*_2_’ ranging from 0 to 1 (perfect specialization), describes the degree of network-level specialization, whereas the standardized Kullback-Leibler distance *d’* describes the degree of species-level specialization^61^. The evenness (*E*_2_, based on Shannon’s index) is a measure of the skewness in the distribution of interaction frequencies^61^. The statistics and mathematics underlying these parameters are well described in ref.^62^. The extreme plant or insect specialization was calculated as the proportion of plant or insect species with only one partner in the network. The cumulative distribution of connectivity (number of links per species) was also explored using the degree distribution function of the bipartite package^63^.

In order to determine whether the network structure differed beyond what would be expected due to network size and marginal abundance distributions, 100 null networks^63^ were generated using the Patefield algorithm implemented in the nullmodel function of the bipartite package^57^. Null networks are made through random sampling of our empirical matrix constraining the marginal totals. In that way, the null matrices contain common and rare species like the empirical ones. All indices cited above were assessed for these 100 null networks and mean and 95% confidence intervals were calculated. Network dimensions and sampling intensity are known to both affect most network metrics^6^. Furthermore, there are far more plant species and interactions in *sp-sp* N_seq_ than in *sp-sp* N_obs_ which preclude any direct comparison between the two matrices. To overcome this problem, a random sampling method was used to generate rarefied *sp-sp* N_seq_ with the same size and sampling intensity as *sp-sp* N_obs_. First only insect and plant species that were found in both matrices were kept, so that the two networks were of the same size. Then the total number of visits the *sp-sp* N_seq_ matrix contains was reduced to that of *sp-sp* N_obs_. In this last step, number of visits in *sp-sp* N_seq_ were randomly drawn from a multinomial distribution with a probability matrix derived from the observed frequency of visits in the original *sp-sp* N_seq_. Thousand rarefied *sp-sp* N_seq_ were generated. For a particular network index (including the slope of the relationship between interaction frequency (*f*) and the number of interacting partners (*S*) for plant and pollinator species), the P-value was set equal to the proportion of values calculated on the rarefied *sp-sp* N_seq_ above or below the value calculated on the original *sp-sp* N_obs_. Then standard Bonferroni corrections for multiple comparisons were applied.

### Data Availability

Nucleotide sequences have been published on GenBank (Accession numbers KU974005-KU974022, KU974024-KU974083).

## Electronic supplementary material

Supplementary information

## References

[1] Biesmeijer JC (2006). Parallel declines in pollinators and insect-pollinated plants in Britain and the Netherlands. Science.

[2] Hegland SJ (2010). How to monitor ecological communities cost-efficiently: The example of plant–pollinator networks. Biol Conserv.

[3] Jordano P (2016). Chasing Ecological Interactions. PLoS Biol.

[4] Vázquez DP (2009). Evaluating multiple determinants of the structure of plant-animal mutualistic networks. Ecology.

[5] Bartomeus I (2013). Understanding linkage rules in plant-pollinator networks by using hierarchical models that incorporate pollinator detectability and plant traits. PLoS ONE.

[6] Blüthgen N (2007). Specialization, constraints, and conflicting interests in mutualistic networks. Cur Biol.

[7] Bascompte J (2003). The nested assembly of plant–animal mutualistic networks. Proc Natl Acad Sci USA.

[8] Jordano P (2003). Invariant properties in coevolutionary networks of plant-animal interactions. Ecol Lett.

[9] Olesen JM (2007). The modularity of pollination networks. Proc Natl Acad Sci USA.

[10] Ollerton J (1996). Reconciling ecological processes with phylogenetic patterns: the apparent paradox of plant-pollinator systems. J Ecol.

[11] Johnson SD, Steiner KE (2000). Generalization versus specialization in plant pollination systems. Trends Ecol Evol.

[12] Waser NM (1996). Generalization in pollination systems, and why it matters. Ecology.

[13] Fenster CB (2004). Pollination syndromes and floral specialization. Annu Rev Ecol Evol Syst.

[14] Bosch J (2009). Plant–pollinator networks: adding the pollinator’s perspective. Ecol Lett.

[15] Widmer A, Cozzolino S, Pellegrino G, Soliva M, Dafni A (2000). Molecular analysis of orchid pollinaria and pollinariaremains found on insects. Mol. Ecol..

[16] Petanidou, T. & Potts, S. G. Mutual use of resources in Mediterranean plant–pollinator communities: How specialized are pollination webs? In: Waser N. M., Ollerton J. editors Plant–pollinator interactions, from specialization to generalization: University of Chicago Press, Chicago, IL. pp. 221–244 (2006).

[17] Gomez JM (2007). Pollinator diversity affects plant reproduction and recruitment: the tradeoffs of generalization. Oecologia.

[18] Vázquez DP, Aizen MA (2003). Null model analyses of specialization in plant–pollinator interactions. Ecology.

[19] Dupont YL (2014). Spatial structure of an individual based plant–pollinator network. Oikos.

[20] Tur C (2014). Downscaling pollen–transport networks to the level of individuals. J Anim Ecol.

[21] King C (2013). Why flower visitation is a poor proxy for pollination: measuring single-visit pollen deposition, with implications for pollination networks and conservation. Meth. Ecol Evol.

[22] Lopezaraiza-Mikel ME (2009). The impact of an alien plant on a native plant-pollinator network: an experimental approach. Ecol Lett.

[23] Galimberti A (2014). A DNA barcoding approach to characterize pollen collected by honeybees. PLoS ONE.

[24] Taberlet P (2012). Environmental DNA. Mol Ecol.

[25] Valentini A, Miquel C, Taberlet P (2010). DNA Barcoding for Honey Biodiversity. Diversity.

[26] De Vere N (2017). Using DNA metabarcoding to investigate honey bee foraging reveals limited flower use despite high floral availability. Sci Rep.

[27] Wilson EE, Sidhu CS, Levan KE, Holway DA (2010). Pollen foraging behaviour of solitary Hawaiian bees revealed through molecular pollen analysis. Mol Ecol.

[28] Richardson RT (2015). Rank-based characterization of pollen assemblages collected by honey bees using a multi-locus metabarcoding approach. App Plant Sci.

[29] Richardson RT (2015). Application of ITS2 metabarcoding to determine the provenance of pollen collected by honey bees in an agroecosystem. App Plant Sci.

[30] Pornon, A. *et al*. Using metabarcoding to reveal and quantify plant-pollinator interactions. *Sci Rep* 27282 (2016). 10.1038/srep27282 PMC4891682 27255732

[31] Vamosi JC, Gong YB, Adamowicz SJ, Packer L (2017). Forecasting pollination declines through DNA barcoding: the potential contributions of macroecological and macroevolutionary scales of inquiry. New Phytol.

[32] Thébault E, Fontaine C (2010). Stability of ecological communities and the architecture of mutualistic and trophic networks. Science.

[33] Bell KL, Loeffler VM, Brosi BJ (2017). An rbcL reference library to aid in the identification of plant species mixtures by DNA metabarcoding. App Plant Sci.

[34] Keller A (2015). Evaluating multiplexed next-generation sequencing as a method in palynology for mixed pollen samples. Plant Biol.

[35] Kraaijeveld K (2015). Efficient and sensitive identification and quantification of airborne pollen using next-generation DNA sequencing. Mol Ecol Resour.

[36] Courtney SP (1982). Pollen carried for long periods by butterflies. Oikos.

[37] Willmer, P. Pollination and floral ecology. Princeton University Press (2011).

[38] Kearns CA (1992). Anthophilous fly distribution across an elevation gradient. Am Midl Nat.

[39] Elberling H, Olesen JM (1999). The structure of a high latitude plant-flower visitor system: the dominance of flies. Ecography.

[40] Delmas CEL (2014). Massive floral display affects insect visits but not pollinator-mediated pollen transfer in Rhododendron ferrugineum. Plant Biol.

[41] Lunau K (1992). Limits of of colour learning in a flower-visiting hoverfly *Eristalis tenax* L. (Syrphidae, Diptera). Eur J Neurosci Suppl.

[42] Sutherland JP (1999). The influence of floral character on the foraging behaviour of the hoverfly. Episyrphus balteatus. Entomol Exp Appl.

[43] Popic TJ, Wardle GM, Davila YC (2012). Flower-visitor networks only partially predict the function of pollen transport by bees. Aust Ecol.

[44] Ballantyne G (2015). Constructing more informative plant–pollinator networks: visitation and pollen deposition networks in a heathland plant community. Proc R Soc B.

[45] Ramos-Jiliberto R (2010). Topological change of Andean plant–pollinator networks along an altitudinal gradient. Ecol Complex.

[46] Dorado J (2011). Rareness and specialization in plant–pollinator networks. Ecology.

[47] Ings TC (2009). Ecological networks – beyond food webs. J Anim Ecol.

[48] Araujo MS (2008). Network analysis reveals contrasting effects of intraspecific competition on individual vs. population diets. Ecology.

[49] Delmas CEL (2016). Pollen transfer in fragmented plant populations: insight from the pollen loads of pollinators and stigmas in a mass-flowering species. Ecol Evol.

[50] Morales CL, Traveset A (2010). Interspecific pollen transfer: magnitude, prevalence and consequences for plant fitness. Crit Rev Plant Sci.

[51] Burkle LA (2016). The beta*-*diversity *of* species interactions: Untangling the drivers of geographic variation in plant*-*pollinator diversity and function across scales. Am J Bot.

[52] Beckerman AP, Petchey OL, Warren PH (2006). Foraging biology predicts food web complexity. Proc Natl Acad Sci USA.

[53] Aizen MA (2016). The phylogenetic structure of plant–pollinator networks increases with habitat size and isolation. Ecol Lett.

[54] Carstensen DW (2014). Beta diversity of plant-pollinator networks and the spatial turnover of pairwise interactions. PLoS ONE.

[55] Memmott J (2007). Global warming and the disruption of plant-pollinator interactions. Ecol Lett.

[56] Marrero HJ (2014). Effect of land use intensification on specialization in plant-floral visitor interaction networks in the Pampas of Argentina. Agri Ecosys Environ.

[57] Faegri, K. & Van Der Pijl, L. The principles of pollination ecology. Pergamon Press (1979).

[58] Pacini E, Hesse M (2005). Pollenkitt–its composition, forms and functions. Flora.

[59] Orford KA, Murray PJ, Vaughan IP, Memmott J (2016). Modest enhancements to conventional grassland diversity improve the provision of pollination services. J. App Ecol.

[60] Dormann CF (2008). Introducing the bipartite Package: Analysing Ecological Networks. R News.

[61] Vázquez DP (2009). Uniting pattern and process in plant – animal mutualistic networks: a review. Ann Bot.

[62] Blüthgen N, Fründ J, Vazquez DP, Menzel F (2008). What do interaction network metrics tell us about specialization and biological traits?. Ecology.

[63] Dormann CF (2009). Indices, graphs and null models: analyzing bipartite ecological networks. Open Eco J.

