# Supplementary material for: DNA metabarcoding data unveils invisible pollination networks
Source: Sci Rep. 2017 Dec 4;7:16828. doi: 10.1038/s41598-017-16785-5 (PMC5715002; doi:10.1038/s41598-017-16785-5)
Supplement: Supplementary file 1 — Supplementary information [file 41598_2017_16785_MOESM1_ESM.pdf]

Supplementary information

DNA metabarcoding data unveils invisible pollination networks

André Pornon[1,2*], Christophe Andalo[1,2], Monique Burrus[1,2], Nathalie Escaravage[1,2]

[1] Laboratoire Evolution and Diversité Biologique EDB, Université Toulouse III Paul Sabatier, F-31062 Toulouse, France ;

[2] CNRS, EDB, UMR 5174, F-31062 Toulouse, France.

andre.pornon@univ-tlse3.fr; christophe.andalo@univ-tlse3.fr;

monique.burrus@univ-tlse3.fr; nathalie.escaravage@univ-tlse3.fr

* Correspondence: andre.pornon@univ-tlse3.fr

Supplementary Fig. S1. Interaction matrices showing plant-pollinator modules obtained from metabarcoding (a) and visit surveys (b).

(a)

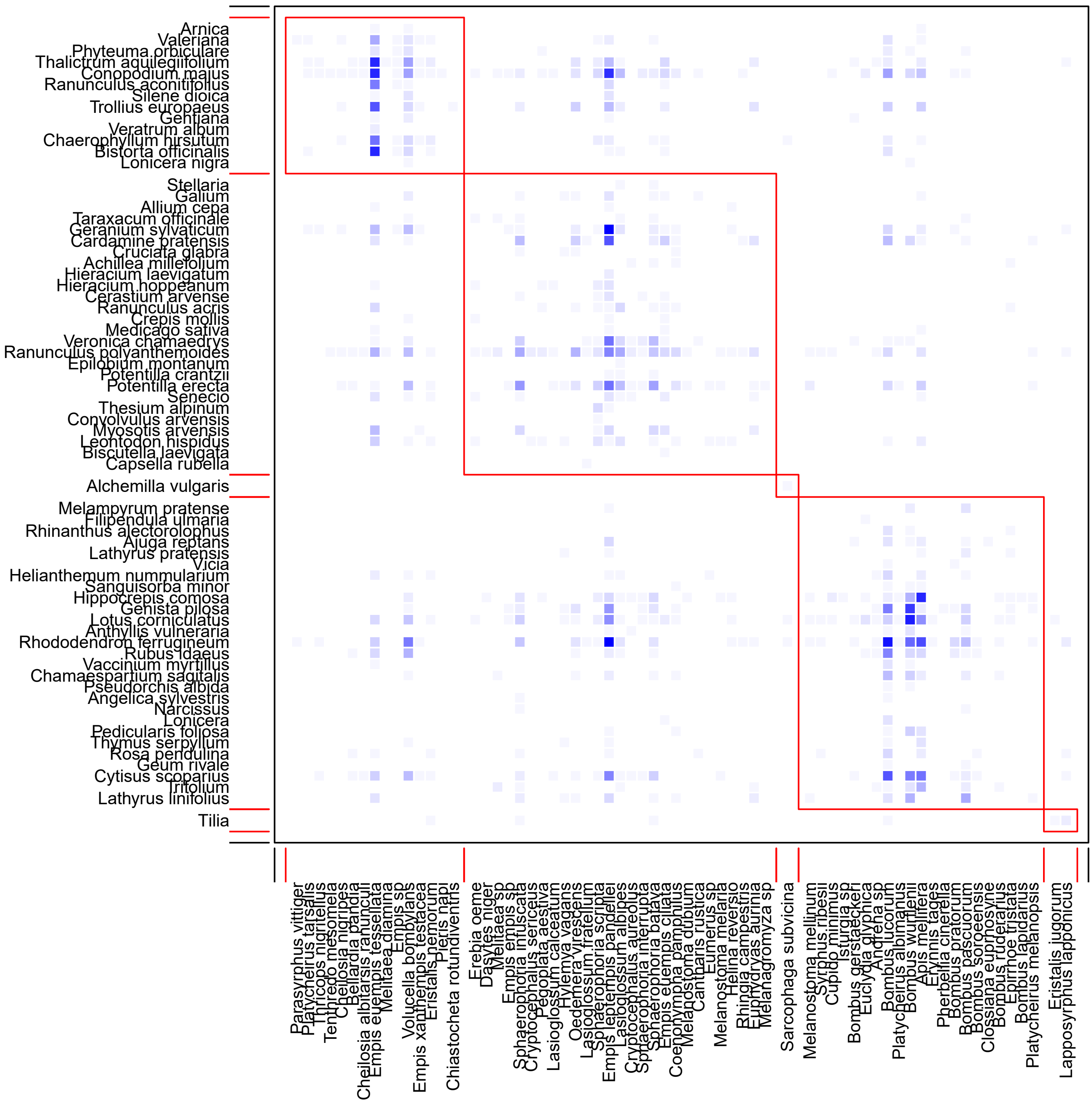

(b)

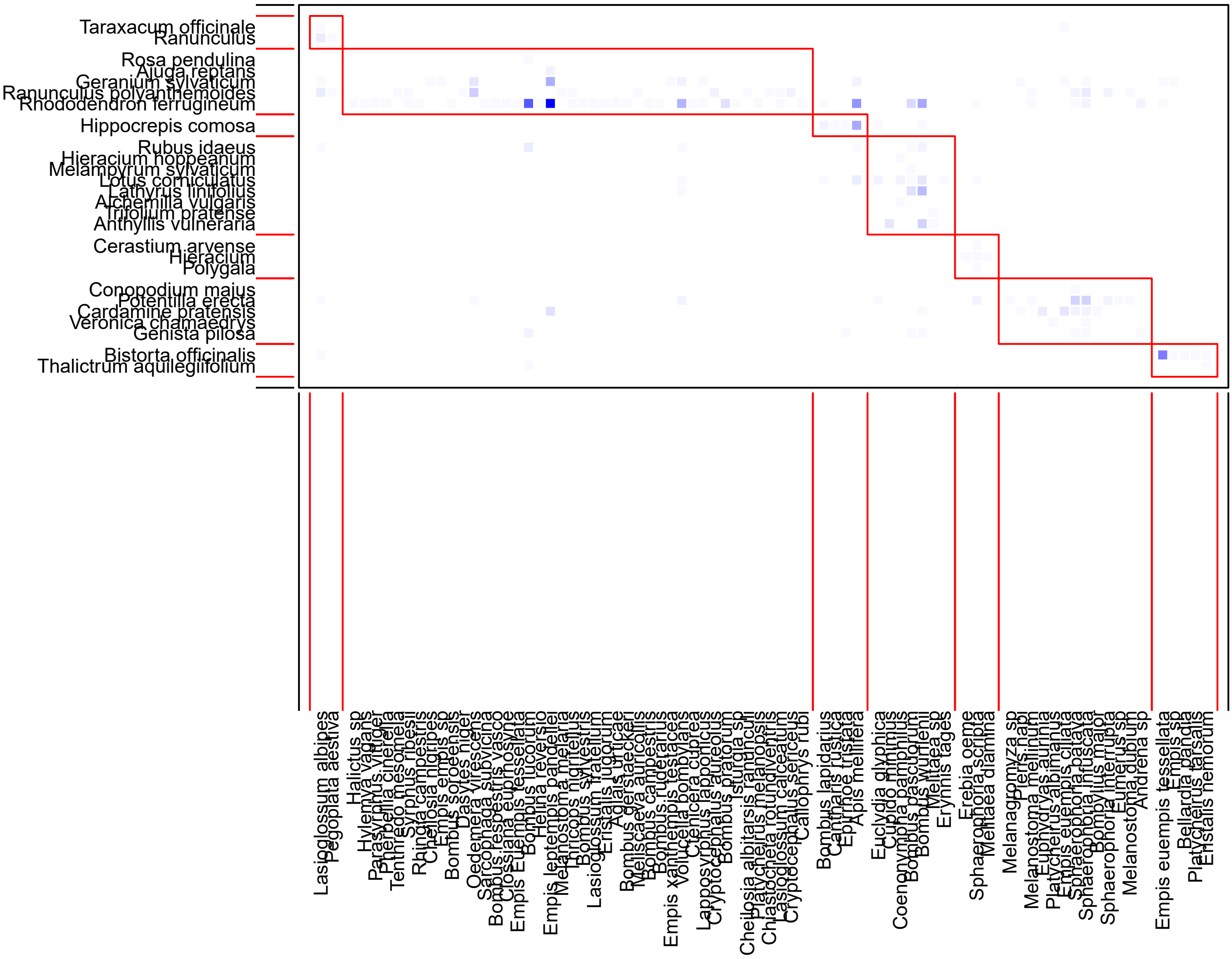
Taraxacum officinale
Ranunculus
Rosa pendulina
Ajuga reptans
Geranium sylvaticum
Ranunculus polyanthemoides
Rhododendron ferrugineum
Hippocrepis comosa
Rubus idaeus
Hieracium hoppeanum
Melampyrum sylvaticum
Lotus corniculatus
Lathyrus linifolius
Alchemilla vulgaris
Trifolium pratense
Anthyllis vulneraria
Cerastium arvense
Hieracium
Polygala
Conopodium majus
Potentilla erecta
Cardamine pratensis
Veronica chamaedrys
Genista pilosa
Bistorta officinalis
Thalictrum aquilegiifolium
Lasioglossum albipes
Pegoplata aestiva
Halictus sp
Hylemya vagans
Parasyrphus vittiger
Pherbellia cinerella
Tenthredo mesomela
Syrphus ribesii
Rhingia campestris
Cheilosia nigripes
Empis empis sp
Bombus soroeensis
Dasytes niger
Oedemera virescens
Sarcophaga subvicina
Bombus respestris vasco
Clossiana euphrosyne
Empis Euempis tessellata
Bombus lucorum
Helina reversio
Empis leptempis pandellei
Melanostoma mellaria
Thricops nigritellus
Bombus sylvestris
Lasioglossum fratellum
Eristalis jugorum
Aglais urticae
Bombus gerstaeckeri
Meliscaeva auricollis
Bombus campestris
Bombus ruderarius
Empis xanthempis testacea
Volucella bombylans
Cetonia cuprea
Lapposyrphus lapponicus
Cryptocephalus aureolus
Bombus pratorum
Isturgia sp
Cheilosia albitarsis ranunculi
Platycheirus melanopsis
Chiastocheta rotundiventris
Lasioglossum calceatum
Cryptocephalus sericeus
Callophrys rubi
Bombus lapidarius
Cantharis rustica
Ephippe tristata
Apis mellifera
Euclydia glyphica
Cupido minimus
Coenonympha pamphilus
Bombus pascuorum
Bombus wurflenii
Melitaea sp
Erynnis tages
Erebia oeme
Sphaerophoria scripta
Melitaea diamina
Melanagromyza sp
Pieris napi
Melanostoma mellinum
Euphydryas aurinia
Platycheirus albimanus
Empis euempis ciliata
Sphaerophoria batava
Sphaerophoria infuscata
Bombylius major
Sphaerophoria interrupta
Eumerus sp
Melanostoma dubium
Andrena sp
Empis euempis tessellata
Empis sp
Bellardia pandia
Platycheirus tarsalis
Eristalis nemorum

Supplementary Fig. S2. Bipartite pollinator individuals-plant species networks built from visit surveys (N_{obs}, right panels) and metabarcoding (Nseq, left panels). Cm : *Conopodium majus*; Rf: *Rhododendron ferrugineum*; Tsp: *Trifolium sp.*; Lc: *Lotus corniculatus*; Cs: *Cytisus scoparius*; Hc: *Hippocrepis comosa*; Ri: *Rubus idaeus*; Gs: *Geranium sylvaticum*; Csa: *Chamaespartium sagitalis*; Gp: *Genista pilosa*; Cp: *Cardamine pratensis*; Rp: *Rosa pendulina*; Ta: *Thalictrum aquilegiifolium*; Ll: *Lathyrus linifolius*; Ms: *Melampyrum sylvaticum*; Av: *Anthyllis vulneraria*; Bo: *Bistorta officinalis*; Ch: *Chaerophyllum hirsutum*; Te: *Trollius europaeus*; Ra: *Ranunculus aconitifolius*; Vo: *Valeriana officinalis*; Rpo: *Ranunculus polyanthemoides*; Vc: *Veronica chamaedrys*; Pe: *Potentilla erecta*; Ar: *Ajuga reptans*; Tal: *Thesium alpinum*; Pv: *Polygala vulgaris*; Hsp: *Hieracium sp.*; Ca: *Cerastium arvense.*

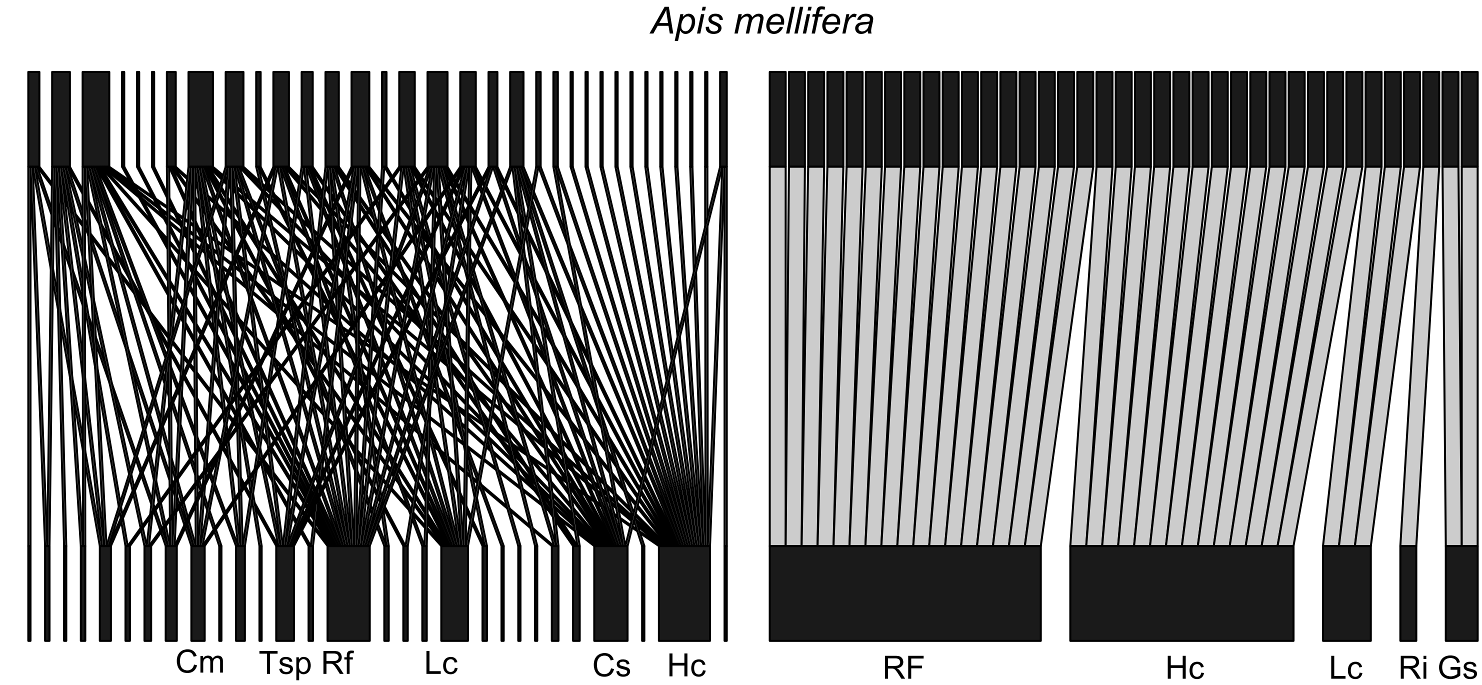

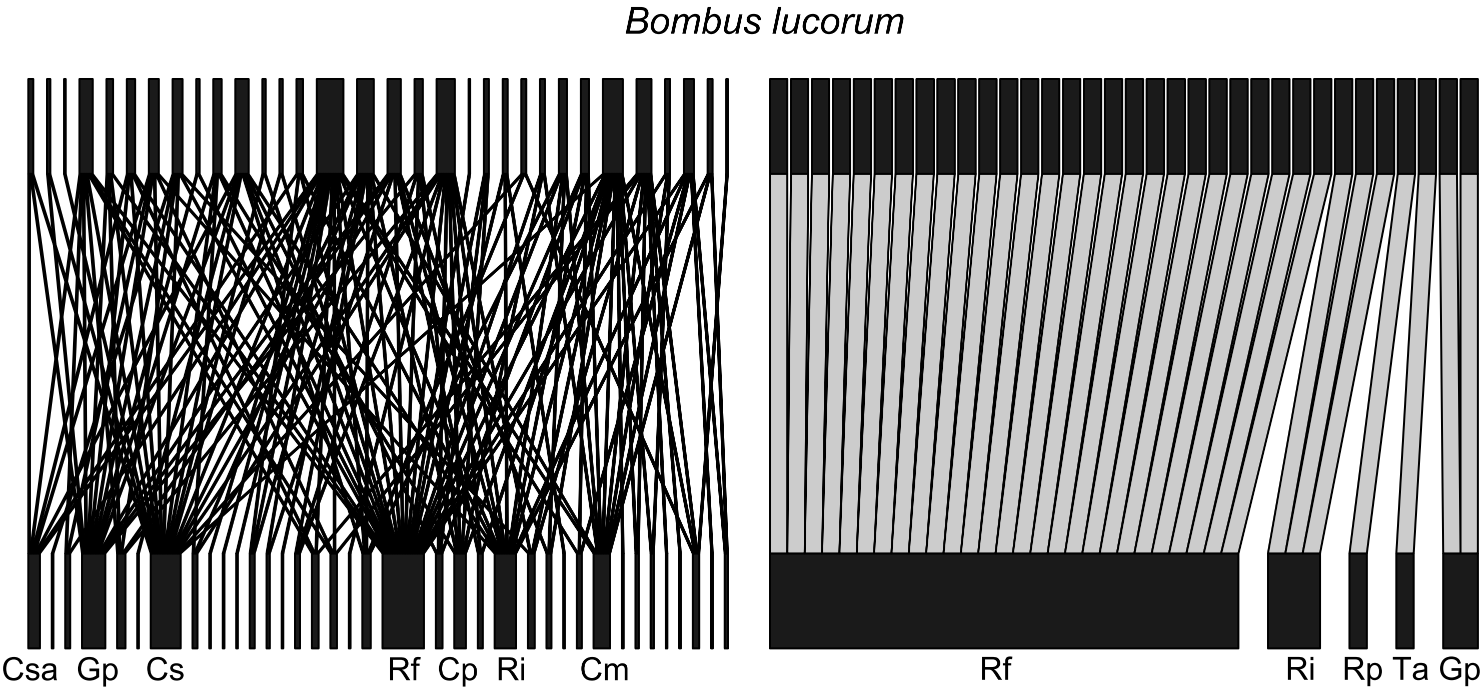

Bombus pascuorum

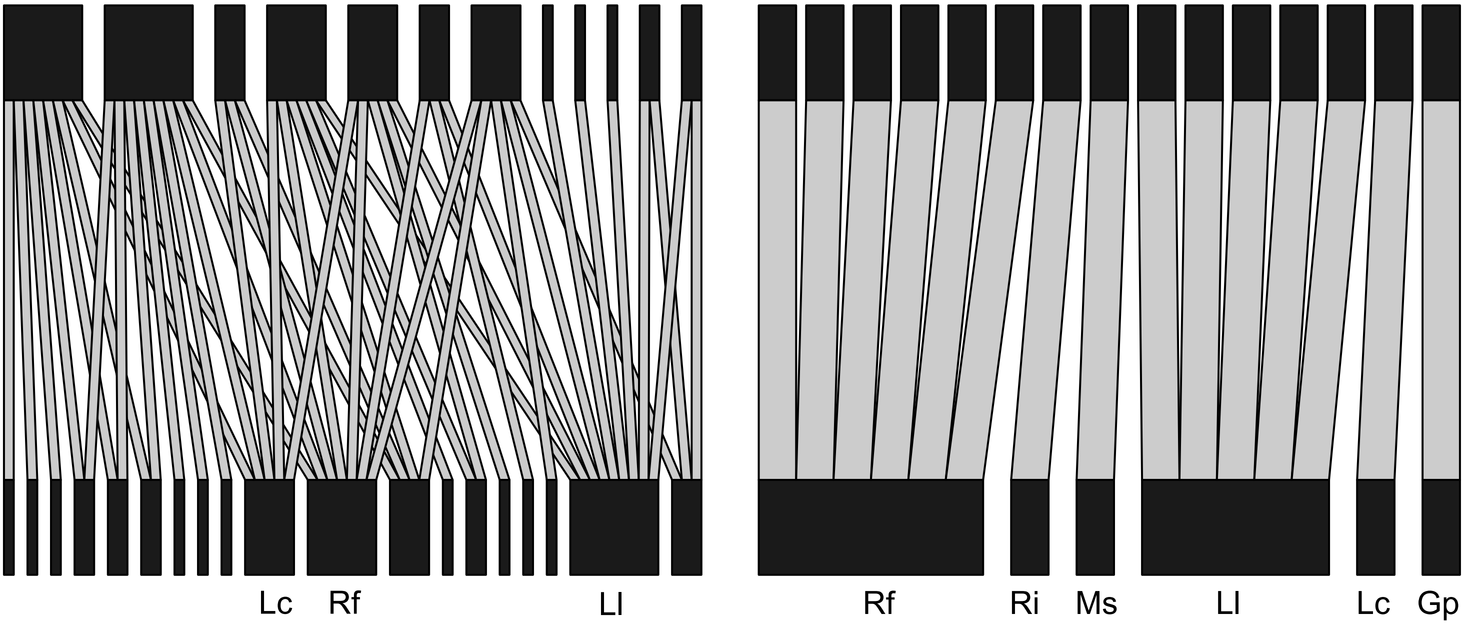

Bombus wurflenii

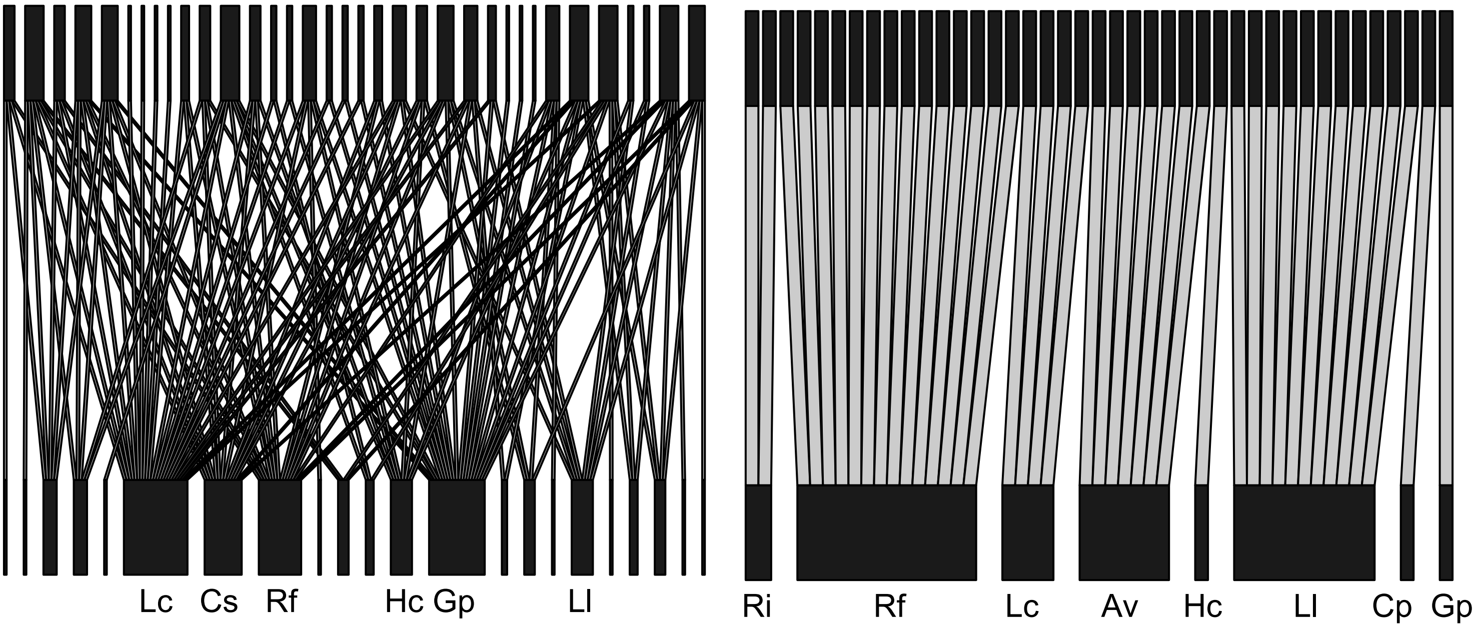

Empis euempis tessellata

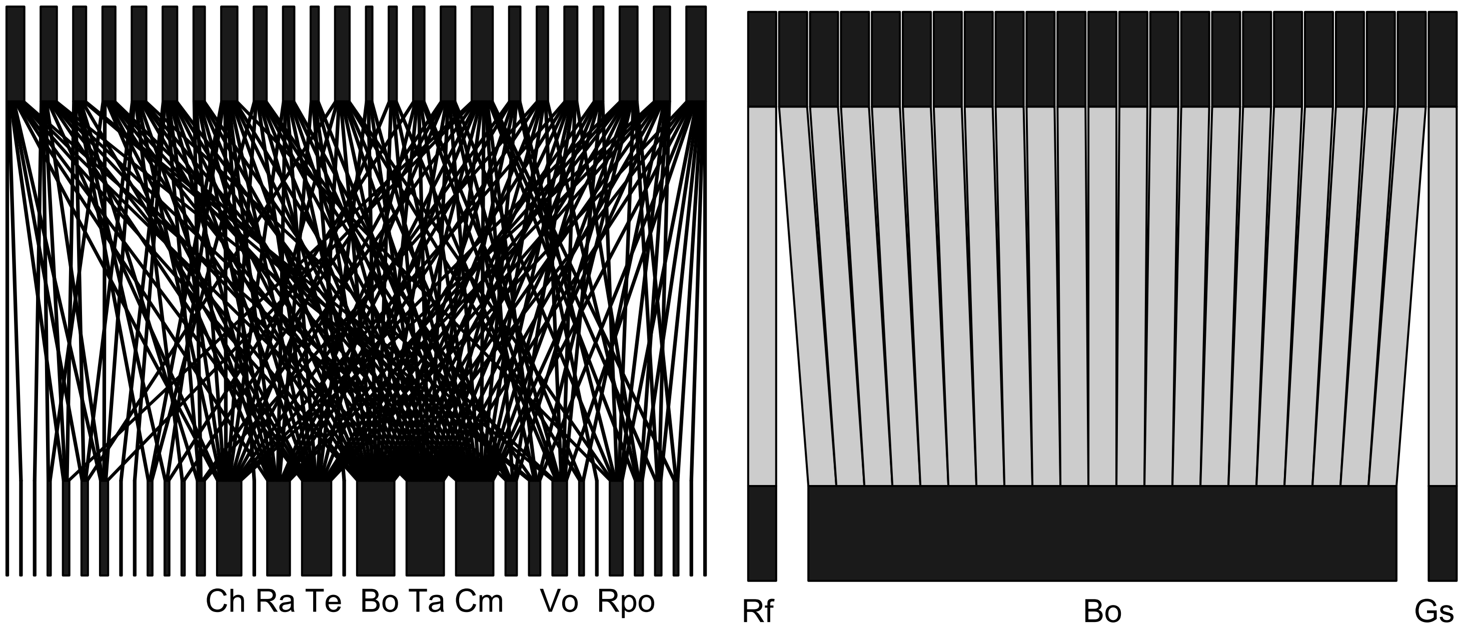

Empis leptempis pandellei

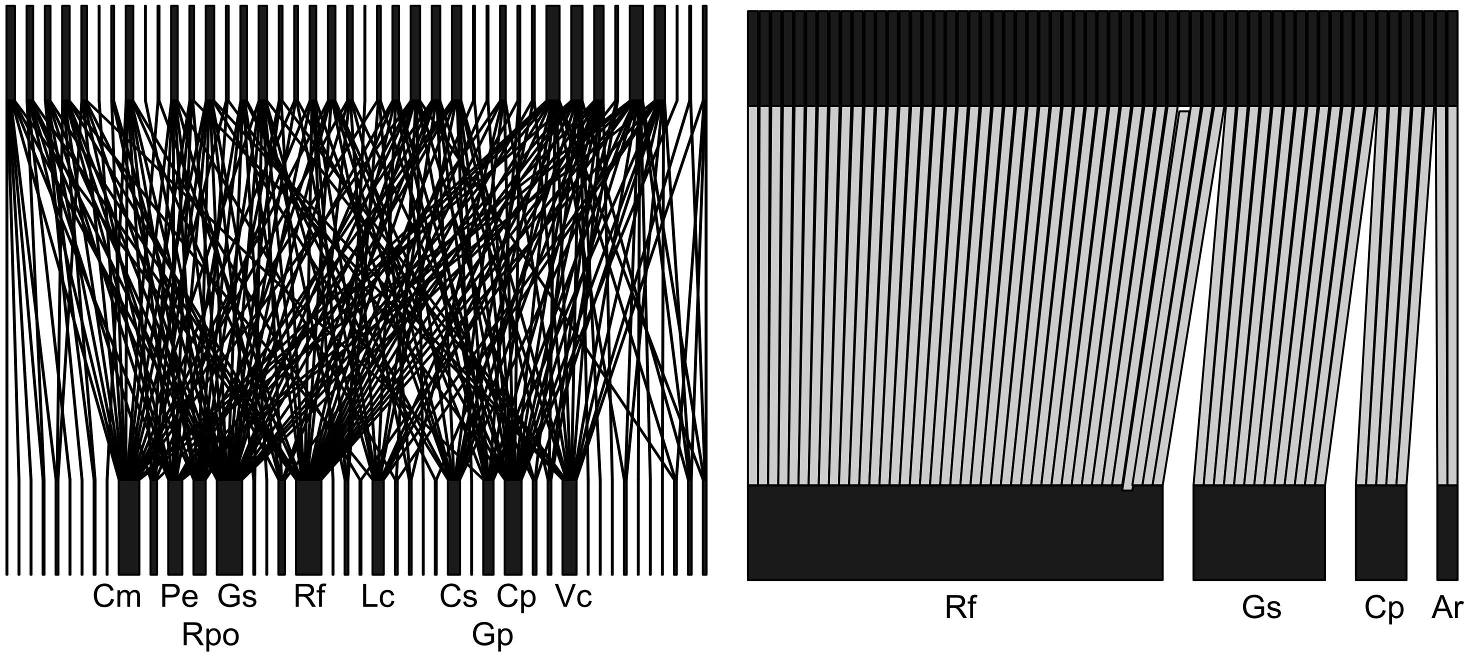

Sphaerophoria batava

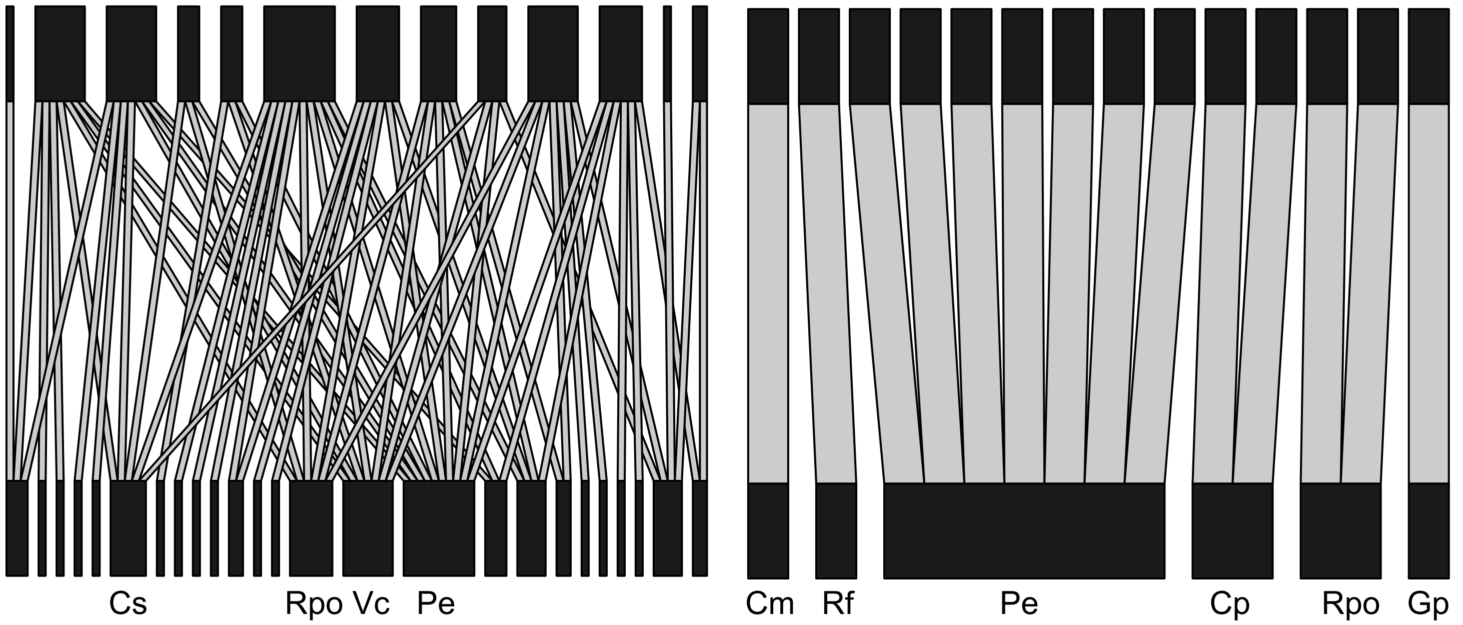

Sphaerophoria infuscata

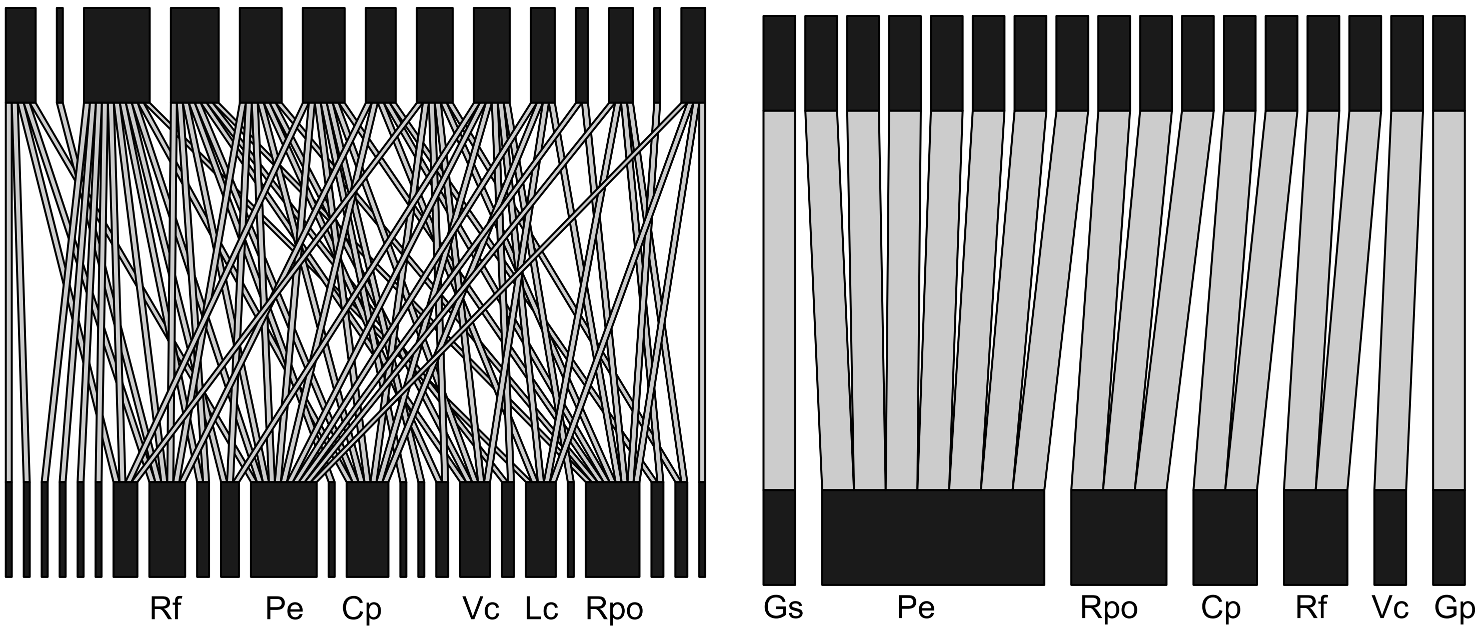

Sphaerophoria scripta

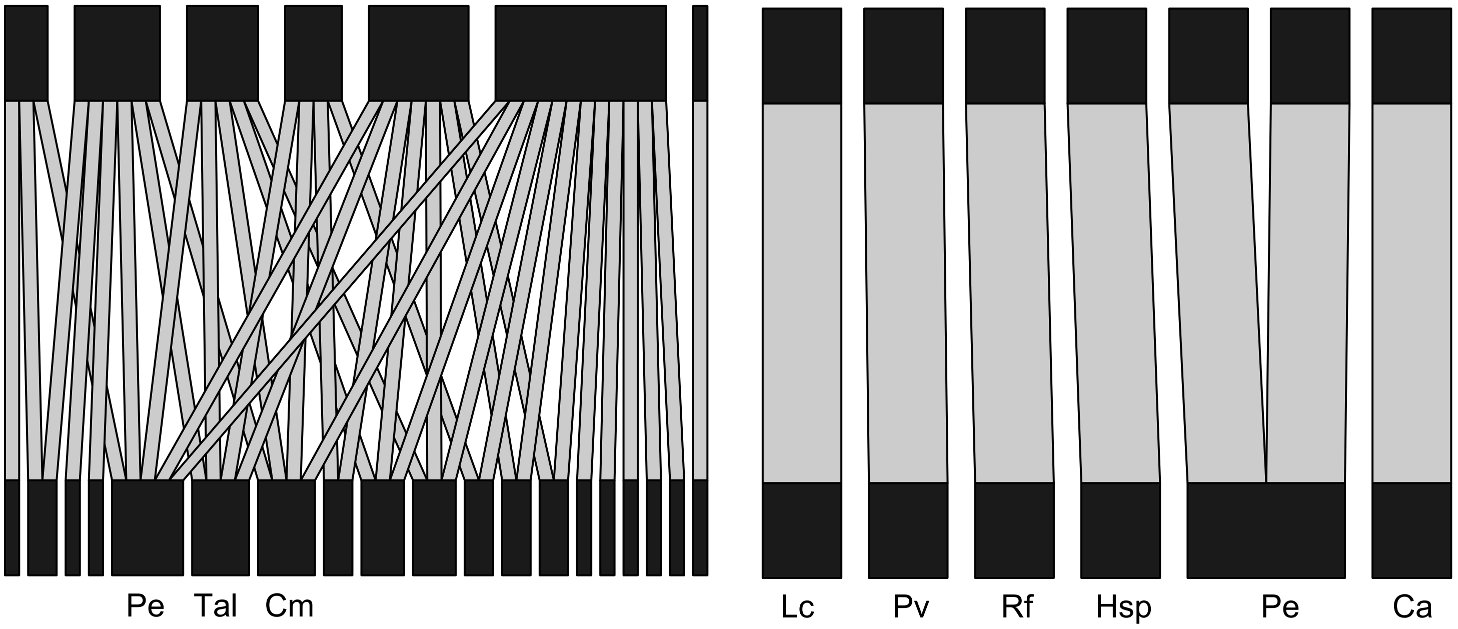

Volucella bombylans

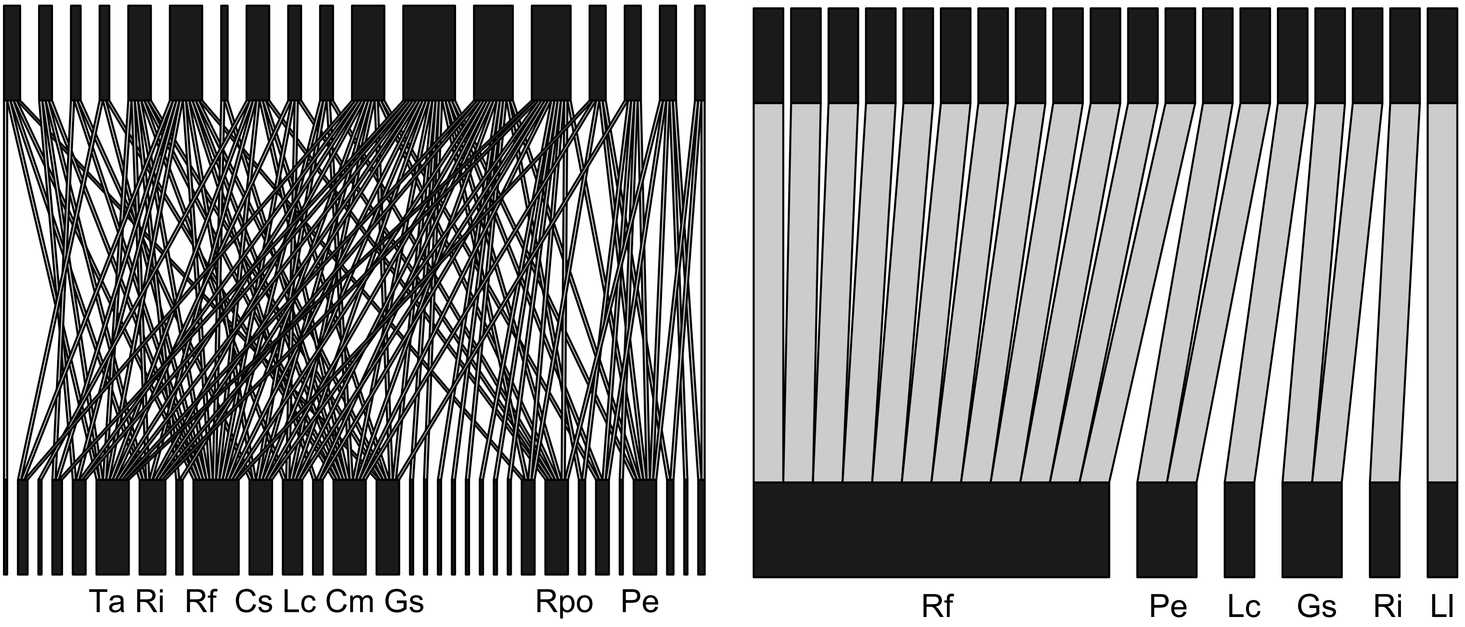

Oedemera virescens

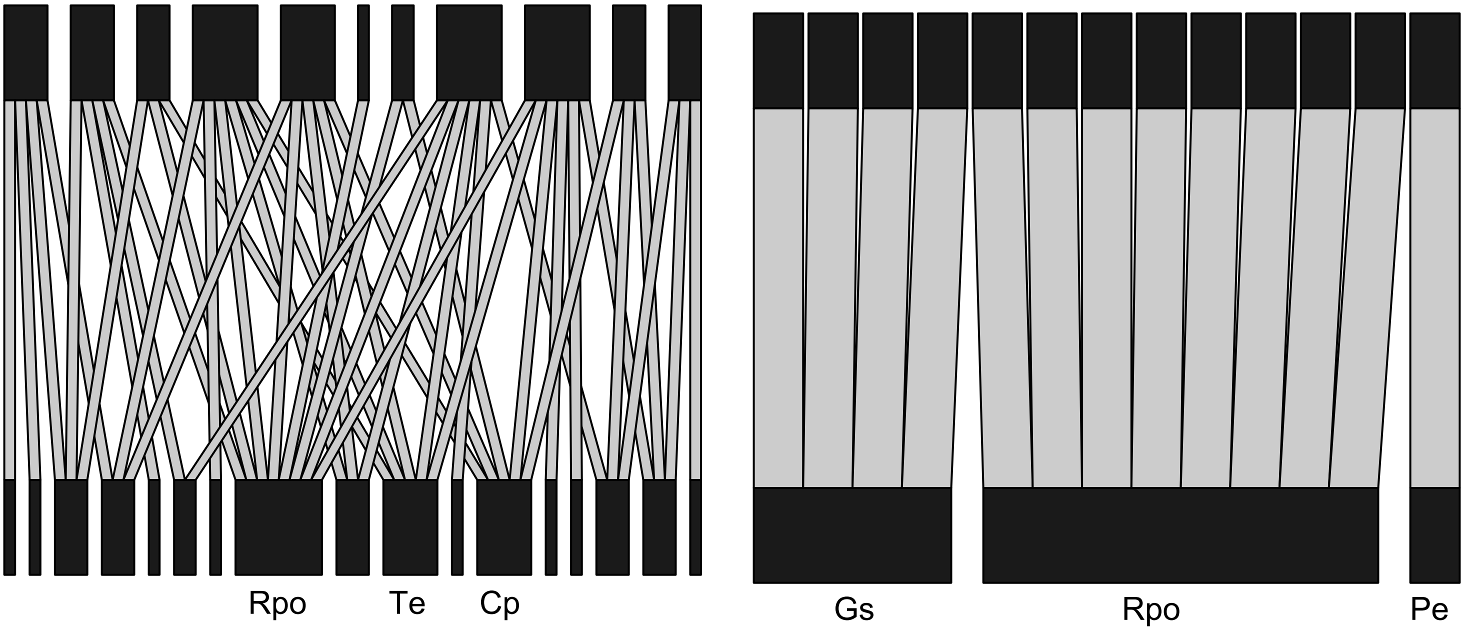

Supplementary Fig. S3. Frequency distribution of samples (insects) according to their number of *Poaceae* sequences. The red line indicates the threshold used and above which a visit was inferred.

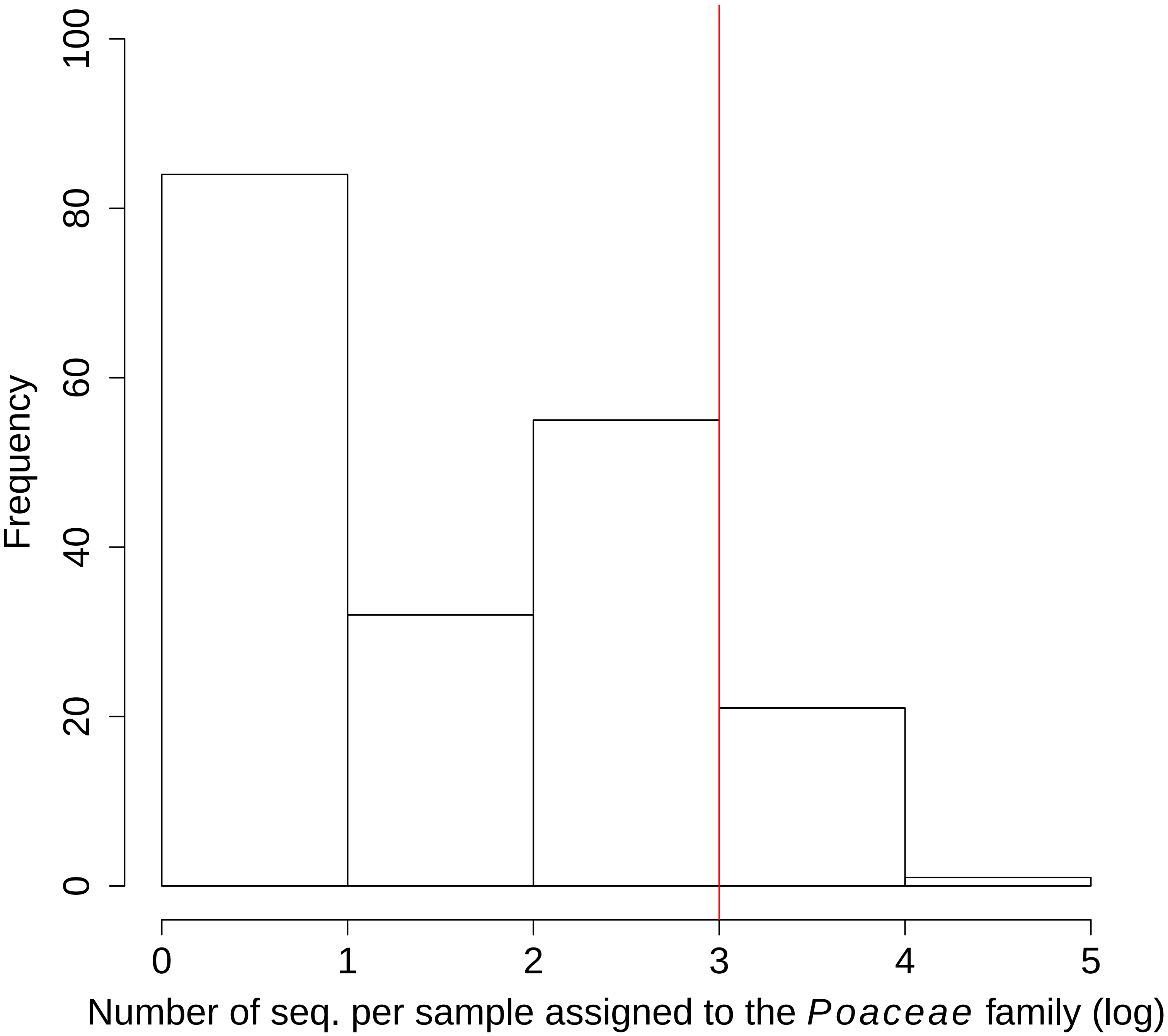

Supplementary Table S1. Characteristics of individual pollinator-plant species networks obtained from visit surveys (N_{obs}) and metabarcoding (N_{seq}). In between brackets are confidence interval values. * indicates that networks differed significantly from their corresponding null models ($P < 0.05$); No.: number.

	A. mellifera		*B. lucorum*		*B. pascuorum*		*B. wurflenii*		*E. e.tessellata*		*E. l. pandellei*	
	N_{obs}	N_{seq}	N_{obs}	N_{seq}	N_{obs}	N_{seq}	N_{obs}	N_{seq}	N_{obs}	N_{seq}	N_{obs}	N_{seq}
No. insect individuals (A)	37	33	34	32	15	12	41	35	23	24	61	41
No. plant species(P)	5	29	5	37	6	19	8	22	3	32	4	43
Network size (AxP)	185	957	170	1184	96	228	328	770	69	768	244	1763
No. interactions (I)	37	131*<br>(100-112)	34	165*<br>(130-146)	15	46*<br>(36-42)	41	129*<br>(101-114)	23	200*<br>(150-166)	61	248*<br>(198-217)
Connectance (C=I/AxP)	0.2	0.14*<br>(0.10-0.12)	0.2	0.14*<br>(0.11-0.12)	0.17	0.20*<br>(0.16-0.18)	0.125	0.17*<br>(0.13-0.15)	0.33	0.26*<br>(0.19-0.22)	0.25	0.14*<br>(0.11-0.12)
Nestedness	0.64	0.933*<br>(0.93-0.86)	0.67	0.899*<br>(0.891-0.798)	0.69	0.865*<br>(0.85-0.69)	0.79	0.9*<br>(0.88-0.81)	0.58	0.74*<br>(0.72-0.61)	0.575	0.90*<br>(0.88-0.835)
Interaction evenness	0.69	0.71*<br>(0.66-0.68)	0.69	0.72*<br>(0.68-0.70)	0.60	0.705*<br>(0.64-0.68)	0.64	0.73*<br>(0.68-0.71)	0.74	0.80*<br>(0.74-0.76)	0.75	0.74*<br>(0.69-0.71)
Interaction density	0.88	2.11*<br>(1.61-180)	0.87	2.39*<br>(1.88-2.12)	0.71	1.48*<br>(1.16-1.35)	0.84	2.26*<br>(1.77-2)	0.88	3.57*<br>(2.68-2.96)	0.94	2.95*<br>(2.36-2.58)
Mean plant linkage level (P/I)	7.4	4.52*<br>(3.45-3.86)	6.8	4.46*<br>(3.51-3.95)	2.5	2.42*<br>(1.89-2.21)	5.125	5.86*<br>(4.59-5.18)	7.67	6.25*<br>(4.68-5.18)	15.25	5.77*<br>(4.6-5.05)
Mean insect linkage level (P/I)	1	3.97*<br>(3.03-3.39)	1	5.16*<br>(4.06-4.56)	1	3.83*<br>(3-3.5)	1	3.68*<br>(2.88-3.26)	1	8.33*<br>(6.25-6.92)	1	6.05*<br>(4.83-5.29)
Interaction diversity (H_2)	NA	0*<br>(0.11-0.22)	NA	0*<br>(0.095-0.172)	NA	0*<br>(0.01-0.26)	NA	0*<br>(0.11-0.21)	NA	0*<br>(0.14-0.20)	NA	0*<br>(0.099-0.17)
Mean plant specialization index *d'*	NA	0.16*<br>(0.24-0.36)	NA	0.19*<br>(0.26-0.34)	NA	0.14*<br>(0.19-0.35)	NA	0.12*<br>(0.21-0.34)	NA	0.09*<br>(0.14-0.20)	NA	0.16*<br>(0.20-0.28)
Mean insect specialization index *d'*	0.14	0.12*<br>(0.25-0.38)	0.16	0.2*<br>(0.28-0.36)	0.30	0.18*<br>(0.29-0.49)	0.22	0.14*<br>(0.22-0.31)	0,09	0.12*<br>(0.22-0.28)	0.14	0.18*<br>(0.25-0.32)
Extreme specialization insect (%)	100	39.4ns<br>(39.4-42.42)	100	9.37ns<br>(9.37-15.6)	100	25ns<br>(25-33.3)	100	20ns<br>(20-28.5)	100	0ns<br>(0-0)	100	9.76ns<br>(9.76-12.19)
Extreme specialization plant (%)	20	31.03ns<br>(31.0-37.9)	40	32.4ns<br>(32.4-35.1)	66.67	52.6ns<br>(52.6-57.9)	37.5	36.4ns<br>(36.4-36.4)	66.67	31.2ns<br>(31.2-34.4)	0	37.21ns<br>(37.21-39.53)
Modularity	NA	0.35*<br>(0.38-0.46)	NA	0.37ns<br>(0.365-0.44)	NA	0.45 ns<br>(0.435-0.54)	NA	0.35*<br>(0.37-0.44)	NA	0.24*<br>(0.28-0.34)	NA	0.32*<br>(0.325-0.38)
No. modules	NA	6ns<br>(5-9)	NA	5ns<br>(5-9)	NA	4ns<br>(4-8)	NA	5ns<br>(5-8)	NA	6ns<br>(5-7)	NA	7ns<br>(5-9)

Continued

	S. batava		*S. infuscata*		*S. scripta*		*V. bombylans*		*O. virescens*	
	N_{obs}	N_{seq}	N_{obs}	N_{seq}	N_{obs}	N_{seq}	N_{obs}	N_{seq}	N_{obs}	N_{seq}
No. insect individuals (A)	**14**	**13**	**17**	**14**	**7**	**7**	**19**	**18**	**13**	**11**
No. plant species(P)	**6**	**25**	**7**	**24**	**6**	**19**	**6**	**31**	**3**	**17**
Network size (AxP)	**84**	**325**	**119**	**336**	**42**	**133**	**114**	**558**	**39**	**187**
No. interactions (I)	**14**	**62*** (50-58)	**17**	**71*** (55-65)	**7**	**38*** (29-35)	**19**	**117*** (93-105)	**13**	**43*** (33-41)
Connectance (C=I/AxP)	**0.17**	**0.19*** (0.15-0.18)	**0.14**	**0.21*** (0.16-0.19)	**0.17**	**0.29*** (0.22-0.26)	**0.17**	**0.21*** (0.17-0.19)	**0.33**	**0.23*** (0.18-0.22)
Nestedness	**0.69**	**0.77ns** (82.2-68.3)	**0.73**	**0.84*** (0.82-0.68)	**0.5**	**0.71 ns** (0.76-0.57)	**0.72**	**0.792*** (0.789-0.65)	**0.50**	**0.73ns** (0.76-0.58)
Interaction evenness	**0.6**	**0.71*** (0.66-0.70)	**0.59**	**0.73*** (0.67-0.71)	**0.52**	**0.74*** (0.67-0.72)	**0.62**	**0.75*** (0.70-0.73)	**0.7**	**0.72*** (0.66-0.71)
Interaction density	**0.7**	**1.63*** (1.32-1.53)	**0.71**	**1.87*** (1.45-1.71)	**0.54**	**1.46*** (1.11-1.35)	**0.76**	**2.39*** (1.90-2.14)	**0.81**	**1.54*** (1.18-1.46)
Mean plant linkage level (P/I)	**2.33**	**2.48*** (2-2.32)	**2.43**	**2.96*** (2.29-2.71)	**1.17**	**2*** (1.53-1.84)	**3.17**	**3.77*** (3-3.38)	**4.33**	**2.53*** (1.94-2.41)
Mean insect linkage level (P/I)	**1**	**4.77*** (3.84-4.46)	**1***	**5.07*** (3.93-4.64)	**1**	**5.43*** (4.14-5)	**1**	**6.5*** (5.17-5.83)	**1***	**3.91*** (3-3.73)
Interaction diversity (H_2)	**NA**	**0*** (0.072-0.24)	**NA**	**0*** (0.084-0.23)	**NA**	**0*** (0.085-0.40)	**NA**	**0*** (0.09-0.19)	**NA**	**0*** (0.06-0.31)
Mean plant specialization index d'	**NA**	**0.17*** (0.18-0.30)	**NA**	**0.15*** (0.19-0.31)	**NA**	**0.165*** (0.169-0.29)	**NA**	**0.17*** (0.21-0.29)	**NA**	**0.145ns** (0.142-0.31)
Mean insect specialization index d'	**0.40**	**0.30*** (0.31-0.49)	**0.40**	**0.215*** (0.28-0.43)	**0.71**	**0.34 ns** (0.31-0.57)	**0.31**	**0.20*** (0.27-0.36)	**0.18**	**0.24*** (0.28-0.45)
Extreme specialization insect (%)	**100**	**15.4ns** (15.4-23.1)	**100**	**14.3ns** (14.3-21.4)	**100**	**14.3ns** (14.3-14.3)	**100**	**0ns** (0-5.55)	**100**	**9.09ns** (9.09-18.2)
Extreme specialization plant (%)	**50**	**56ns** (56-64)	**42.9**	**45.8ns** (45.8-54.2)	**83.3**	**47.4ns** (47.4-63.2)	**50**	**38.7ns** (38.7-41.9)	**33.33**	**47.1ns** (47.1-52.9)
Modularity	**NA**	**0.40*** (0.43-0.54)	**NA**	**0.36*** (0.39-0.49)	**NA**	**0.38*** (0.42-0.55)	**NA**	**0.33*** (0.35-0.43)	**NA**	**0.42ns** (0.416-0.57)
No. modules	**NA**	**5ns** (5-8)	**NA**	**5ns** (4-8)	**NA**	**5ns** (4-6)	**NA**	**5ns** (5-7)	**NA**	**6ns** (4-6)

Supplementary Table S2. Characteristics of species-based networks built with metabarcoding data and the application of a 500 *vs.* 2000 sequence threshold (see Methods). CI: confidence interval. * indicated that either the 500 seq. or 2,000 seq. *sp-sp* N_{seq} differed significantly from its corresponding null models ($P \leq 0.01$). T: temperature.

Sequence threshold	500 seq.	95% CI	2000 seq.	95% CI
No. insect species (A)	66		65	
No. plant species (P)	71		65	
Network size (A x P)	4686		4225	
No. links (I)	749*	791-825	484*	538-566
Connectance (C=I/AxP)	0.16*	0.169-0.176	0.11*	0.127-0.134
Nestedness (100-T)/100	0.9ns	0.89-0.93	0.94ns	0.95-0.92
Modularity (M)	0.26*	0.11-0.13	0.316*	0.135-0.167
Number of modules	6ns	4-6	5ns	4-7
Interaction density I/(A+P)	5.47*	5.77-6.02	3.72*	4.14-4.35
Mean plant linkage level (I/P)	10.55*	11.14-11.62	7.45*	8.28-8.71
Mean insect linkage level (I/A)	11.35*	11.98-12.5	7.45*	8.28-8.71
Interaction diversity (H_2')	0.15*	0.081-0.098	0.22*	0.091-0.111
Interaction evenness ($E_2 = H_2'/H_{max}$)	0.71*	0.73-0.735	0.69*	0.704-0.711
Mean plant specialization index *d'*	0.19*	0.12-0.16	0.24*	0.145-0.206
Mean insect specialization index *d'*	0.155ns	0.14-0.18	0.20ns	0.152-0.206
Extreme plant specialization (%)	7.04ns	5.63-8.45	16.92ns	13.85-16.93
Extreme insect specialization (%)	6.06ns	6.06-7.57	12.31ns	12.31-15.38
